# Gray whale habitat use and reproductive success during seismic surveys near their feeding grounds: comparing state-dependent life history models and field data

**DOI:** 10.1007/s10661-022-10024-9

**Published:** 2022-10-18

**Authors:** Lisa Schwarz, Elizabeth McHuron, Marc Mangel, Glenn Gailey, Olga Sychenko

**Affiliations:** 1 Institute of Marine Sciences, University of California, Santa Cruz, CA 95064 USA; 2 Department of Ecology and Evolutionary Biology, University of California, Santa Cruz, CA 95064 USA; 3 Ocean, and Ecosystem Studies, Cooperative Institute for Climate, University of Washington, Seattle, WA 98195 USA; 4 Theoretical Ecology Group, Department of Biology, University of Bergen, 9020 Bergen, Norway; 5 Puget Sound Institute, University of Washington, Tacoma, WA 98402 USA; 6 Cetacean EcoSystem Research, Lacey, WA 98516 USA

**Keywords:** Bayesian analysis, *Eschrichtius robustus*, Russia, Population consequences of disturbance, PCOD, Stochastic dynamic programming

## Abstract

We used a stochastic dynamic programming (SDP) model to quantify the consequences of disturbance on pregnant western gray whales during one foraging season. The SDP model has a firm basis in bioenergetics, but detailed knowledge of minimum reproductive length of females (*L*_min_) and the relationship between length and reproductive success (*R*_fit_) was lacking. We varied model assumptions to determine their effects on predictions of habitat use, proportion of animals disturbed, reproductive success, and the effects of disturbance. Smaller *L*_min_ values led to higher predicted nearshore habitat use. Changes in *L*_min_ and *R*_fit_ had little effect on predictions of the effect of disturbance. Reproductive success increased with increased *L*_min_ and with higher probability of reproductive success by length. Multiple seismic surveys were conducted in 2015 off the northeast coast of Sakhalin Island, with concomitant benthic prey surveys, photo-identification studies, and whale distribution sampling, thus providing a unique opportunity to compare output from SDP models with empirical observations. SDP model predictions of reproductive success and habitat use were similar with and without acoustic disturbance, and SDP predictions of reproductive success and large-scale habitat use were generally similar to values and trends in the data. However, empirical estimates of the proportion of pregnant females nearshore were much higher than SDP model predictions (a large effect, measured by Cohen’s *d*) during the first week, and the SDP model overestimated whale density in the south and underestimated density around the mouth of Piltun Bay. Such differences in nearshore habitat use would not affect SDP predictions of reproductive success or survival under the current seismic air gun disturbance scenario.

**Supplementary information:**

The online version contains supplementary material available at 10.1007/s10661-022-10024-9.

## Introduction

Each summer and fall, western gray whales (*Eschrichtius robustus*) forage off the NE Sakhalin coast in Russia in two defined areas, known as the offshore and nearshore feeding areas, both of which are close to long-term oil and gas developments and their associated activities. In 2015, two oil and gas companies conducted multiple seismic surveys near or overlapping those gray whale feeding areas, which could change gray whale behavior and distribution (Gailey et al., 2007, 2016; Muir et al., 2015, 2016; Yazvenko et al., 2007). Specifically, there was concern that the combined 2015 seismic surveys could lead to decreased gray whale foraging activity and reduce population growth (Aerts et al., 2022).

**Fig. 1:**
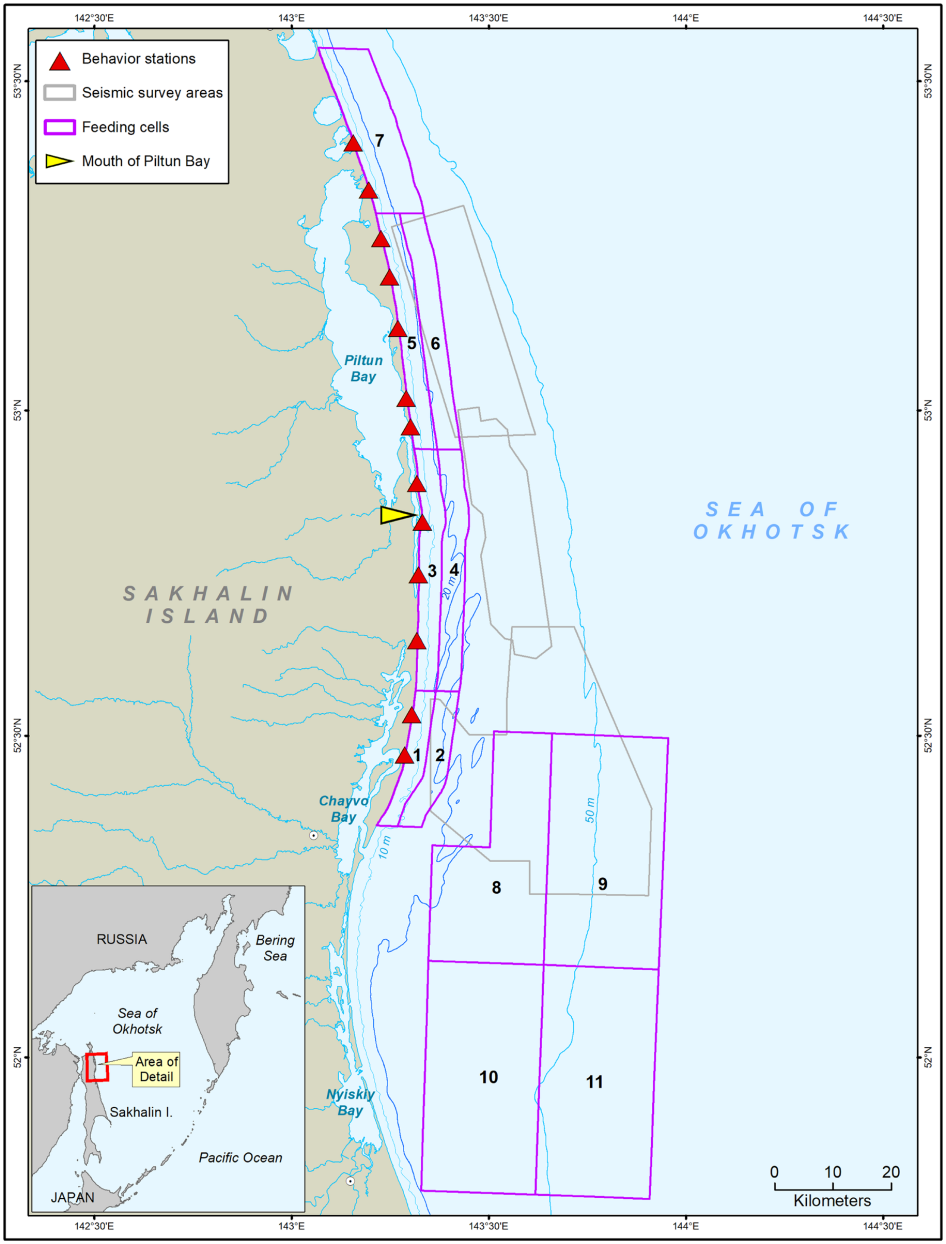
Map showing the defined feeding cells (numbered pink polygons) and the 2015 seismic survey areas (gray polygons). Distribution data were collected at the 13 shore-based behavior stations (red triangles), and nearshore gray whale density was highest around the mouth of Piltun Bay (tip of yellow triangle)

The population-level effects of previously documented changes in behavior and distribution remain unclear. Since they are capital breeders, gray whales would generally be less affected by lost foraging opportunities than income breeders (McHuron et al., 2017). However, several characteristics of the population make it susceptible to decline. Their two known feeding areas are small compared to that of eastern gray whales and many other baleen whales (Mate et al., 2011; Moore et al., 2003), and they exhibit a high degree of site fidelity to these areas (Fig. 1) (Bröker et al., 2020). In addition, the estimated population size is small for a K-selected species, with 175 identified non-calf individuals, of which 33 are known reproducing females (Cooke et al., 2017). Furthermore, environmental factors such as ice conditions can limit the duration of their foraging season, reduce energy intake, and lower calf survival (Bradford et al., 2012; Gailey et al., 2020; Perryman et al., 2020).

The population consequences of disturbance (PCOD) framework offers a conceptual model linking environmental impacts, changes in behavior, physiological health, and population growth through a series of transfer functions (New et al., 2014). Stochastic dynamic programming (SDP) modeling provides a way to simulate natural situations of disturbance in the presence of environmental variation while allowing individuals to vary in their behavioral responses to maximize reproductive fitness, ultimately linking behavior to individual health and health to demographic rates (Clark & Mangel, 2000; Houston et al., 2006; Mangel & Clark, 1988; Mangel & Ludwig, 1992). Although the application of SDP models to determine the effects of reduced marine mammal foraging is relatively new, the modeling approach has already provided novel insights on whale populations (Costa et al., 2016; McHuron et al., 2017; Pirotta et al., 2019).

McHuron et al. (2021), using hypothetical acoustic disturbance data, developed the first SDP model for pregnant western gray whales. They showed that disturbances that excluded or reduced foraging in the nearshore area, where prey energy density is relatively low, did not affect predictions of female survival or reproductive success (carrying a fetus to term and weaning the calf back on the foraging grounds). Regardless of disturbance scenarios, access to the high-energy, amphipod-dense offshore feeding area was particularly important to successful reproduction (McHuron et al., 2021). However, the effects of important underlying model assumptions about minimum maternal length and the reproductive fitness functions were not investigated.

In 2015, benthic prey surveys, photo-identification studies, and distribution investigations occurred in conjunction with seismic surveys and intensive acoustic monitoring in and near the gray whale feeding areas, providing a unique opportunity to further develop, apply, and test the SDP model of McHuron et al. (2021). Specifically, the objectives of the work described here were to (1) perform sensitivity analyses on the western gray whale SDP model of McHuron et al. (2021) to determine how changes in minimum reproductive female length and the reproductive fitness function affect predictions of habitat use, probability of disturbance, and reproductive success; (2) compare disturbance-free SDP model predictions of habitat use and reproductive success with predictions from models that include measured acoustic exposure during the 2015 seismic surveys; and (3) compare SDP model predictions that account for actual acoustic disturbance and the observed gray whale distribution and photo-identification data collected in 2015.

## Methods

### Study area

Following McHuron et al. (2021), we divided the feeding area into 11 cells (seven nearshore and four offshore) based on the 2015 seismic survey locations, gray whale density, and the locations of long-term benthic sampling stations (Fig. 1). Overall, prey energy density is much higher and a higher proportion of energy is provided by amphipods in the offshore feeding area (Maresh et al., 2022). Young of the year only utilize the nearshore area, and as they age, gray whales tend to increase their use of the offshore area (Schwarz et al., 2022).

### SDP model

The SDP modeling development involves two parts, backward iteration and forward simulation, as described in detail in McHuron et al. (2021). Backward iteration determines the behavioral choices that maximize reproductive fitness using prey availability and energy requirements for survival and reproduction. Forward simulation predicts how a population will respond based on individuals’ behavioral choices, prey availability, and disturbance.

Because they are capital breeders, pregnant gray whales on the foraging grounds have to procure sufficient energy to meet metabolic and reproductive needs for the foraging period, subsequent migrations, and time at the breeding grounds. The bulk of reproductive costs (most of gestation and almost all of lactation) are incurred during migrations and in the breeding area, when females are presumed to fast or have minimal foraging success (Nerini, 1984; Sanchez-Pacheco et al., 2001). McHuron et al. (2021) relied on quantification of metabolic rates of gray whales, cost of pregnancy, and cost of lactation from two previous bioenergetics modeling efforts (Villegas-Amtmann et al., 2015, 2017). During foraging, energy allocation was prioritized in the following order: current metabolic needs, current fetal growth, future metabolic needs, and future gestation and lactation. If a foraging female could not meet her current energetic needs, fat reserves were used to maintain metabolism as well as allow for fetal growth at a reduced rate. Otherwise, surplus energy was stored as fat for future needs. The survival probabilities of the female and fetus during the non-foraging period were a function of the size of the fetus and the female’s total stored fat energy when the female left the foraging ground. Smaller females could store less fat for use during the fasting period.

Given the fetus length and the female’s fat energy stores at the end of the foraging season, fitness values of all potential behaviors at previous time steps were calculated via backwards iteration (Clark & Mangel, 2000; Mangel & Clark, 1988), thereby determining the behavior that optimized reproduction. At each daily time step, females could (1) leave the foraging area completely, (2) continue to feed at the current location, (3) travel within the current cell, or (4) travel to a different cell. In the modeling work described here, we used backward iteration to determine the probability of a whale selecting one of the four behavioral options given her fat mass, the length of the fetus, location, and food availability at each time step.

We calculated available benthic prey energy for each of the 11 cells (Fig. 1) for three periods (early, mid, and late season) using biomass values and energy density of six benthic prey groups collected in 2015 (Maresh et al., 2022). For use in the SDP model, prey energy distribution probabilities were modeled as lognormal distributions for each cell and time period and converted to equally spaced, discrete categories (McHuron et al., 2021).

In the forward Monte Carlo simulation, individual females behaved in one of the four ways at each 6-h time step. The behavioral state was determined using the probability of each behavior state conditioned on the female’s fat mass, the length of her fetus, location, and food availability. Behavioral state probabilities were determined from the backward iterations. We drew both population and individual parameters at the start of the feeding period from appropriate distributions (described below). The method allows us to capture uncertainty and natural variability in parameter estimates, including population size, body length of each female, starting fat mass, and starting fetal length. We also varied travel (speed, time, and linearity) and foraging parameters (length of dive cycle and percentage of time spent diving) for each individual and 6-h time step by drawing from appropriate distributions.

We assumed females died if their fat mass fell below 5% of length-specific total body mass based on studies from many mammalian species and cited in McHuron et al. (2021). Survival of a female to the following foraging season was a function of her length and fat reserves when she left the foraging grounds. Alternatively, a female could die at any time from random chance based on the probability of mortality for non-calves estimated in mark-recapture studies (Cooke et al., 2017). Because the SDP model of McHuron et al. (2021) considered only a single breeding event, we did not include the option of abortion during a foraging season, which would occur in a multi-year SDP model (e.g., McHuron et al., 2018). We follow the terminology of Cooke (2010) and define reproduction (or reproductive success) as successfully carrying a fetus to term and weaning the calf upon return to the foraging grounds.

We slightly modified the forward simulation by changing the arrival date of individuals to reflect a later arrival at the foraging grounds distributed over several days (normal distribution with a mean date of June 15 ± 5 days s.d.). The modification does not change overall survival or reproductive rates because females in the model compensate for lost foraging at the beginning of the season (McHuron et al., 2021), but the more dispersed, later arrival date better matches field observations. The model structure allowed females to arrive in the feeding area as early as mid-May and forage for up to 25 weeks.

For each population replicate, we calculated proportions of pregnant females in the nearshore feeding area (Cells 1–7), the offshore feeding area (Cells 8–11), or outside both areas (not arrived yet or left the area) over the foraging season for each 6-h bin. To determine the mean and variance of those proportions over time, 6-h bins of the calculated proportions were pooled by the three areas, population replicate, and week of year. Weekly time bins were used to reduce the effects of individual movements that created a high level of stochasticity at smaller time scales.

### Objective 1: Application of SDP model with different reproductive female lengths and fitness functions

**Fig. 2:**
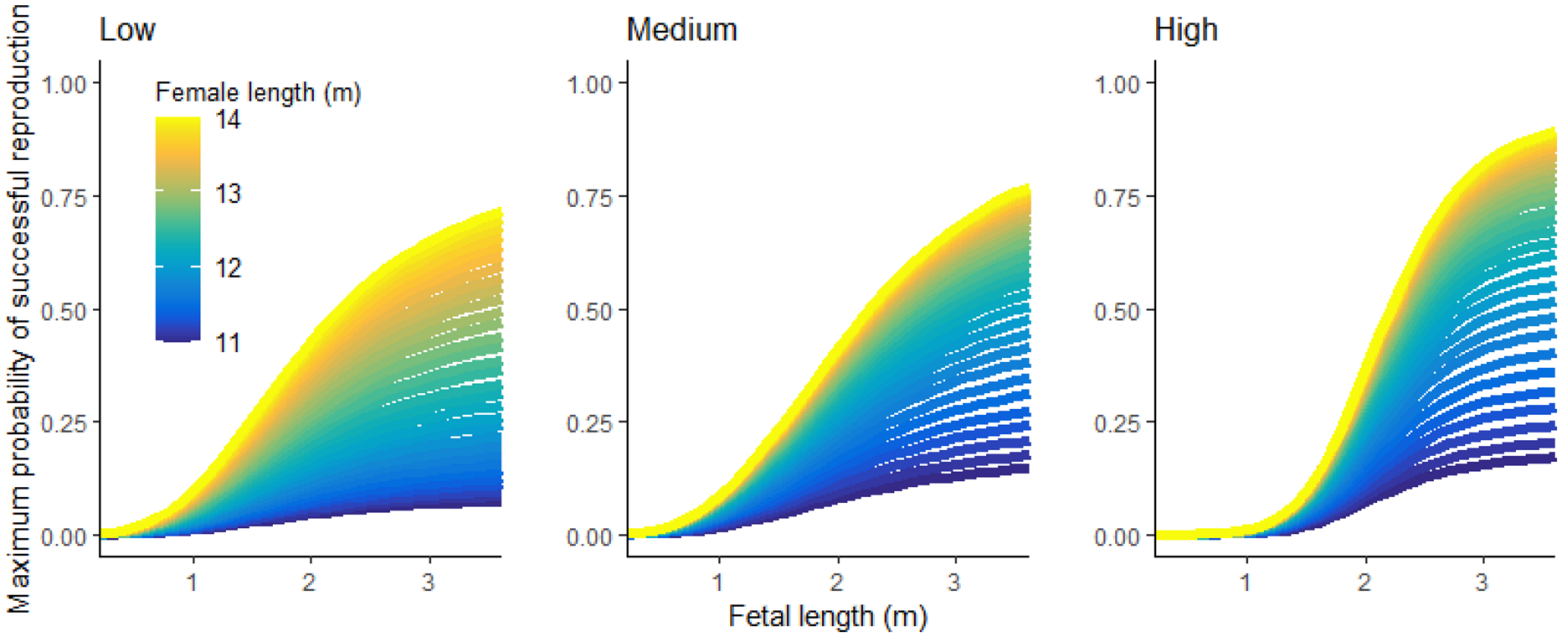
Maximum probability of successful reproduction as a function of female length and fetal length for the three tested fitness functions. Values indicate the fitness function for maximum possible maternal fat mass on the last possible day of foraging

The probability of successful reproduction (reproductive fitness function; *R*_fit_) is dependent on time of departure from the foraging grounds, maternal mass at departure, maternal length, and fetal length at departure. Because *R*_fit_ is based on functions with a high level of uncertainty, we tested three different fitness functions to determine how the shape of the function affected overall reproductive success and female behavioral choices, as indicated by differences in foraging location over time. We used (1) *R*_fit_ of McHuron et al. (2021), (2) *R*_fit_ with a higher probability of calf survival by maternal mass and a small change in the shape of the function defining calf survival vs. fetus length, and (3) *R*_fit_ with a higher probability of calf survival by maternal mass and a large change in the shape of the function defining calf survival vs. fetus length (Supp. Mat.: Variations in *R*_fit_; Table S1; Figs. S1 and S2). We refer to the fitness functions as low, medium, and high probability of reproductive success (Fig. 2).

McHuron et al. (2021) sampled female body length from a normal distribution (12.7 ± 0.6 m) with a range limit of 11–14 m. That distribution was based on lengths of southbound pregnant females from whaling data (Rice & Wolman, 1971; Villegas-Amtmann et al., 2015). However, their SDP model results indicated smaller females are unlikely to successfully reproduce. In addition, the length distribution of reproductive females in the field is most likely shifted higher compared to the distribution tested by McHuron et al. (2021) because photo-identification studies can only identify a reproductive female once she has successfully returned to the foraging grounds with a calf, perhaps not when she was first pregnant. Thus, for comparison with field data, three additional distributions of reproductive female length were tested in this paper. These length distributions had the same normal mean and standard deviation as in McHuron et al. (2021) but limited the minimum reproductive lengths (*L*_min_) to 12.1, 12.7, or 13.0 m instead of 11.0 m. The lower limits were determined by estimating the length at which 50% of the females had successfully reproduced, based on recent growth rate information of gray whale females (Agbayani et al., 2020) and the three *R*_fit_ (Supp. Mat.: Determination of minimum limit on reproductive female length; Fig. S3).

To provide a distribution of resulting reproductive rates from forward simulations, 100 populations were simulated for each of the twelve model permutations, that is, for three fitness (*R*_fit_) functions with four minimum reproductive lengths (*L*_min_) without disturbance.

The proportion of females nearshore and offshore was very similar for many of the twelve models. Therefore, to compare SDP model predictions of habitat use at a finer scale of SDP cell, we focused on a representative subset of three models: two models illustrating the highest and lowest nearshore proportions and one model characterizing a roughly average nearshore proportion. On a weekly scale, we used Cohen’s *d* (Cohen, 1977; White et al., 2014) to compare results between SDP models. Cohen’s *d* is calculated as the difference in mean predicted values divided by the pooled standard deviation. The magnitude (effect size) of Cohen’s *d* values is defined as little-to-no effect (|*d*|< 0.2), small effect (0.2 ≤|*d*|< 0.5), intermediate effect (0.5 ≤|*d*|< 0.8), and large effect (|*d*|≥ 0.8).

### Objective 2: Comparison of modeled habitat use and reproductive success without disturbance and with measured acoustic disturbance in 2015

Sound levels within and outside both foraging areas during the 2015 seismic surveys were recorded by 40 Autonomous Underwater Acoustic Recorders (AUARs) at 48 locations during the season (Rutenko et al., 2022). Not all locations had recorders for the entire season, with some recorders moved to different locations relative to the seismic activity at the time. The measured air gun sound levels from the AUARs were used to calibrate acoustic propagation models (Rutenko et al., 2022). For the nearshore area, the maximum sound pressure level was estimated in 1-km^2^ blocks over 5 min periods. A block was considered disturbed if the maximum SPL exceeded 163 dB re 1 µPa^2^ mean square sound pressure level (SPL). For the offshore area, the estimation of acoustic metrics was performed on a broader spatial scale of 10 × 10 km (or 100 km^2^) blocks because model calibration was based on records from only one AUAR. Although vessel sounds are known to cause gray whale behavioral and distribution changes (Gailey et al., 2022a, b), for simplicity they were not included in model iterations. Onshore pile driving also occurred in 2015, but it produced localized, low sound levels; like vessel sounds, pile driving sounds were not included in model iterations.

Forward simulations that include disturbance require quantification of the probability of exposure to a disturbance, the probability animals will respond to the disturbance, and the behavioral change that will occur if they do respond. For all twelve SDP models, air gun sound levels recorded during the 2015 seismic surveys were used to quantify disturbance. We computed the probability of exposure to air gun sounds in each SDP cell as the proportion of acoustic blocks above the disturbance threshold in a given SDP cell during each 6-h bin.

We set the threshold for behavioral change at 163 dB re 1µPa^2^ SPL, which has been used for mitigation purposes when minimizing disturbance to gray whales from air gun sounds in the nearshore feeding area (Aerts et al., 2022). The 163 dB re 1µPa^2^ SPL threshold was based on a study in the Bering Sea, which estimated that 10% of gray whales stopped feeding and moved away from the area when exposed to received air gun sound levels of 163 dB re 1µPa^2^ SPL (Malme et al., 1988). We conservatively assumed any whale exposed to ≥ 163 dB re 1µPa^2^ SPL during a 6-h period responded by moving to the next closest undisturbed cell and lost the opportunity to forage for 6 h.

To compare disturbed versus undisturbed SDP model estimates of reproduction, the proportion of pregnant females that successfully reproduced (gave birth and returned to the foraging area the following spring with a calf) was pooled by population replicate for each of the twelve SDP model combinations of the three fitness functions and the four minimum reproductive lengths.

We used Cohen’s *d* as described above to compare disturbed versus undisturbed SDP habitat use predictions (proportion of pregnant females in a cell) for the three representative subsets of SDP models over the field season. We also calculated the predicted proportion of animals disturbed in each 6-h bin using all three SDP models.

### Objective 3: Comparison of model results with observed gray whale distribution and photo-identification data from 2015

During the 2015 foraging season, the most coastal nearshore cells (Cells 1, 3, 5, and 7) were monitored for gray whales via photo-identification and distribution surveys (shore-based scan sampling) on temporal and spatial scales that can be compared with SDP model results. Photo-identification data provided information about individual use of the entire nearshore area over the season (seen versus not seen), including specific determination of nearshore use by known reproductive females. Distribution data, collected using shore-based scan sampling techniques, can determine the spatial occurrence of animals within the nearshore area.

Schwarz et al. (2022) provide details on the photo-identification data collection process off Sakhalin Island. In brief, unique permanent body pigmentation patterns along with additional scarring and barnacle patches were used to identify individual gray whales. Data were collected using a combination of up to five stationary and two mobile shore-based teams as well as up to two inflatable boats. Although effort was high, environmental conditions prevented consistency in photo-identification data collection both temporally and spatially.

Distribution data were collected at hourly intervals to quantify gray whale occurrence patterns within the nearshore area throughout the season, which were compared with SDP model density predictions. Distribution data were collected at 13 shore-based stations by 13 observation teams using scan sampling techniques. Shore-based stations were strategically placed roughly 10 km apart that covered around 122 km of coastal habitat. A synchronized scan was conducted hourly (scan survey), weather permitting, at each station by two observers, equipped with 7 × 50 Fujinon FMTRC-SX reticle binoculars, that continuously scanned from the northern part of their observation area to the southern extent at a constant rate of 9.33°/min. For each gray whale(s) sighted during the scan, the magnetic bearing, reticle, and number of individuals were recorded. Environmental conditions (visibility, Beaufort sea state, swell height, etc.) were recorded prior to conducting a scan. All sighting data were recorded using the Pythagoras software system that calculates the geographic position of the animal using Lerczark and Hobbs’ distance approximation equation combined with Leaper and Gordon’s refraction correction (Gailey & Ortega-Ortiz, 2002; Leaper & Gordon, 2001; Lerczak & Hobbs, 1998). Further methodological details can be found in Gailey et al. (2022b).

We compared SDP model predictions with field estimates of (1) the proportion of pregnant females identified nearshore across the foraging season, (2) pregnant female whale density in the seven nearshore cells over the foraging season, and (3) reproductive rates. Sightings of all possibly pregnant females in 2015 were used in our analysis. The group consisted of known pregnant females that were seen with calves in 2016 and potentially pregnant females that were seen with a calf prior to 2015. Since females known to have successfully reproduced (seen with a calf in 2016) may utilize the habitat differently than other reproductive females, the “known pregnant” or “successfully pregnant” group was also analyzed separately. “All potentially pregnant” females included all successfully pregnant and possibly pregnant females. Note that we analyzed habitat use for a subset of females that were seen in 2015 and 2016, since not all mothers were identified. Also, nearshore counts of pregnant females may be underestimated as photo-identification efforts were largely from shore, and within the nearshore area, older whales tend to forage farther from the coast where identification is not possible (Sychenko, 2011).

To determine the proportion of reproductive females seen in the nearshore area over time (*p*_near_), the number of identified known pregnant or all potentially pregnant females identified nearshore was counted on a daily scale and divided by the total number in their respective group (*N* = 8 and 18, respectively). Zero-inflated beta regression was used to estimate *p*_near_ over time to allow for a daily proportion of zero (no whales of interest) nearshore for a given day. The regression estimates the probability of *p*_near_ > 0 (*α*_*B*_) using the total number of days when photo-identification surveys occurred (*S*) and the number of days when at least one pregnant whale was seen (*S*_y≠0_). Proportions greater than zero were then estimated from a beta distribution, leading to an overall likelihood of

$$\begin{aligned}{bi}_{0}\left({p}_{near}|{\alpha }_{B},{\mu }_{B},\varphi \right)= \left\{\begin{array}{c}1-{\alpha }_{B} \\ {\alpha }_{B}\,f\left({p}_{near}|{\mu }_{B},\varphi \right)\end{array}\right.\begin{array}{c}\;\mathrm{if}\;{p}_{near}=0\\ \mathrm{if} \;0<{p}_{near}<1\end{array},\;\mathrm {where}\end{aligned}$$

$$f\left({p}_{near}|{\mu }_{B},\varphi \right)\sim Beta\left({\mu }_{B},\varphi \right) and {S}_{{y}_{p} \ne 0}\sim Bin\left(S,{\alpha }_{B}\right)$$

Both *α*_*B*_ and the mean of the beta distribution (*µ*_*B*_) were independently estimated as a function of visibility, Beaufort sea state, week of year (week), and week of year squared (week^2^). The beta distribution precision parameter (*φ*) was estimated as a constant. Daily visibility and Beaufort sea states were calculated as the mean of daily station mean values from the 13 shore-based stations, where environmental data were collected during hourly scan samples (Gailey et al., 2022b). Regression results were compared with SDP model predictions of *p*_near_ pooled weekly for Cells 1, 3, 5, and 7 from the disturbance scenario.

To estimate whale density within each of the seven nearshore SDP cells, we converted spatially detailed density estimates from scan surveys (*D*). Methodology for determining detailed density estimates can be found in Gailey et al. (2022b) and Muir et al. (2015). In brief, the nearshore area was divided into a grid of 1-km^2^ blocks. For a given survey, gray whale sightings were assigned to a respective block and converted to density, accounting for the proportion of the block area covered and whale availability (not all whales are above water during a scan). Previous analyses suggest a flat detection probability curve equal to 1.0 out to the maximum distance analyzed (which is unique for each scan station height at 0.1 binocular reticles), so detection probability was set at 1.0 (Muir et al., 2016). Sightings from only one station were used to calculate density when scan stations had overlapping visible area.

To calculate density within the seven large nearshore SDP cells, each 1-km^2^ block was assigned a cell based on its location. Blocks that overlapped cell boundaries were split into two blocks with smaller areas. Survey (*i*), block (*j*), and cell (*k*) specific density estimates (${D}_{\mathrm{i},\mathrm{j},\mathrm{k}}$) were reconverted to count estimates ${N}_{\mathrm{i},\mathrm{j},\mathrm{k}}$ by multiplying by a block’s area (${A}_{\mathrm{j}}$). Counts within a cell were summed over all blocks in that cell and divided by the total block area in that cell (*A*_i,k_) covered during the survey to determine gray whale density in a cell for a given survey $\left({D}_{\mathrm{i},\mathrm{j},\mathrm{k}}\right)$

$$\begin{array}{l}{D}_{i,k}= \frac{\sum_{j=1}^{{J}_{i,k}}{N}_{i,j,k}}{{A}_{i,k}},\;\mathrm{where}\\{N}_{i,j,k}= {D}_{i,j,k}\cdot {A}_{i,j,k}\\{A}_{i,k}=\sum_{j=1}^{{J}_{i,k}}{A}_{i,j,k}\;\mathrm{and}\\{J}_{i,k}=\mathrm{total\;number\;of\;blocks\;in\;survey}\;i\;\mathrm{for\;cell}\;k.\end{array}$$

We used a hurdle gamma regression model to analyze density as a function of time to allow for a density of zero (no whales). The hurdle gamma regression estimates the probability of the density being greater than zero (*α*_*G*_) using the total number of surveys (*R*) and the number of surveys when at least one whale was seen (${R}_{\widehat{\mathrm{D}}\ne 0}$). Densities greater than zero were then estimated from a gamma distribution, leading to an overall likelihood of

$$\begin{aligned}{Gamma}_{0}\left(D|{\alpha }_{G},{\mu }_{G},\omega \right)= \left\{\begin{array}{c}1-{\alpha }_{G} \\ {\alpha }_{G}\,f\left(D|{\mu }_{G},\omega \right)\end{array}\right.\begin{array}{c}\mathrm{if}\;D=0\\ \mathrm{if}\;D>0\end{array},\; \mathrm{where}\end{aligned}$$

$$f\left(D|{\mu }_{G},\omega \right)\sim Gamma\left({\mu }_{G},\omega \right)\;\mathrm{and}\; {R}_{D \ne 0}\sim Bin\left(R,{\alpha }_{G}\right)$$

Both *α*_*G*_ and the mean of the gamma distribution (*µ*_*G*_) were independently estimated as a function of week, week^2^, and week^3^ separately by cell. Not all blocks were observed during every survey, so the proportion of each cell covered in a survey was also included as a predictor variable in the models. The gamma distribution shape parameter (*ω*) was assumed constant.

The above density estimates include all nearshore gray whales, while the SDP predicts density for pregnant whales only. To convert regression estimates to pregnant female density, we used photo-identification data to calculate the proportion of pregnant females (*D*_preg_) compared to the total number of identified whales in the coastal nearshore cells (*p*_preg_) on a daily scale for both known pregnant females and all potentially pregnant females. Logistic regression was used to estimate *p*_pre*g*_ as a function of week, week^2^, daily Beaufort sea state, and visibility. Weekly estimates of *D* were multiplied by estimates of *p*_preg_ and compared with SDP predictions of *D*_preg_ in the nearshore (Cells 1–7) from the three SDP models with disturbance. SDP predictions of *D*_preg_ were calculated as the predicted proportion of females in a cell, multiplied by the number of known pregnant females (or all potentially pregnant females), and divided by total cell area.

The Bayesian R package “brms” was used to estimate posterior distributions of regression parameters and to determine the best-fitting models (Bürkner, 2017, 2018; Plummer, 2019). Priors on parameters were uniform with boundaries wide enough that posterior distributions of the parameters were not truncated at any prior boundaries. Standard practices (multiple independent chains with low lag-1 autocorrelation) ensured mixing, convergence, and stationarity in posterior samples using R packages “MCMCvis,” “coda,” and “bayesplot” (Gabry & Mahr, 2021; Gabry et al., 2019; Plummer et al., 2006; Youngflesh, 2018). Bayesian model stacking using the leave-one-out criteria was used to average over multiple models when no one individual model was weighted > 0.9 (Yao et al., 2017). When Beaufort sea state or visibility was included as a predictor variable, the dependent variable was estimated using posterior samples of parameters and ideal environmental conditions (Beaufort = 0 and visibility = 1). In posterior estimates of density, the proportion of each cell covered in a survey was held constant at the seasonal mean value.

The reproductive success predictions from the twelve SDP model scenarios (with disturbance) were compared with the number of calves identified in 2016 taking into account the number of potentially pregnant females in 2015, defined as known reproductive females that were not observed with a calf in 2015. The reproductive success estimate assumes the inter-birth interval is at least 2 years (Rice & Wolman, 1971; Weller et al., 2009), and all known reproductive females not identified with a calf in 2015 were pregnant. Not all mothers were identified in 2015 and 2016. Nine out of the 11 calves (82%) in 2015 were identified with their mothers, while 8 out of 14 calves (57%) were identified with their mothers in 2016.

As described above, we used Cohen’s *d* (Cohen, 1977; White et al., 2014) to compare SDP model results with and without disturbance. We also used Cohen’s *d* to compare SDP model predictions with estimates derived from field data. The proportion of pregnant females nearshore over the season was compared between SDP predictions and results from regression analysis of photo-identification data on pregnant and potentially pregnant females. Within the nearshore habitat, whale density by cell was compared between SDP predictions and results from regression analysis of empirical whale densities, scaled to pregnant female whale density.

## Results

### Objective 1: Application of SDP model with different reproductive female lengths and fitness functions

**Fig. 3:**
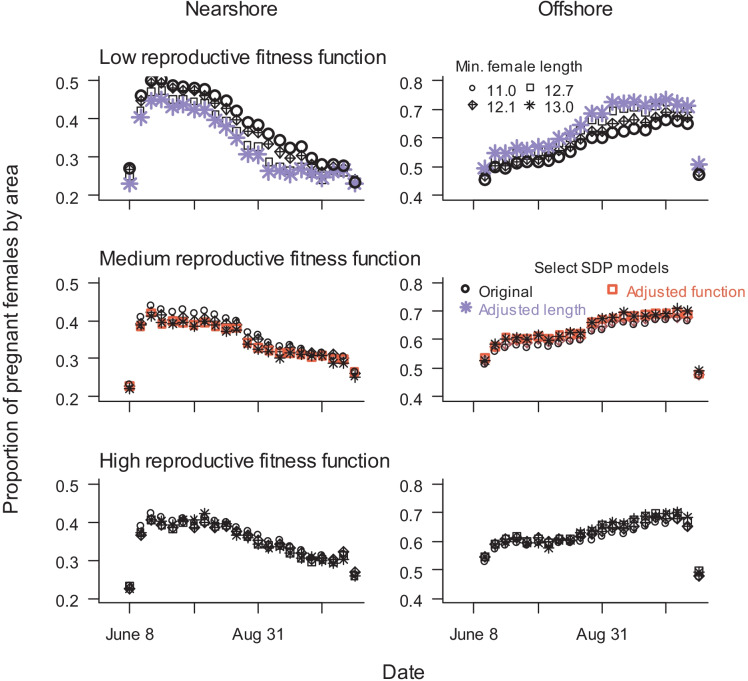
SDP model results of mean weekly proportion of pregnant females nearshore (left column) and offshore (right column) without disturbance, using three different reproductive fitness functions and four minimum maternal lengths. Three combinations of reproductive fitness function and minimum maternal length were used in analyses of the consequences of disturbance: Original = low fitness function, minimum length = 11.0 (bold black circles), adjusted length = low fitness function, minimum length = 13.0 (bold purple stars), and adjusted fitness = medium fitness function, minimum length = 12.7 (bold orange squares)

The SDP model results indicated a relatively equal proportion of the pregnant female population occupying the nearshore and offshore areas (*p*_near_ and *p*_off_, respectively) at the beginning of the foraging season (Fig. 3). The models predicted that *p*_near_ would steadily decline throughout the season, and *p*_off_ would show an opposite trend. The low reproductive fitness function produced higher *p*_near_ predictions earlier in the season compared to the medium and high reproductive fitness functions. Predictions of *p*_off_ were higher with increased minimum maternal length (*L*_min_), although differences in habitat use for different *L*_min_ values became less pronounced with higher reproductive fitness functions. In fact, *L*_min_ did not affect habitat use with the high reproductive fitness function. Using the low reproductive fitness function with the shortest *L*_min_ (11.0 m) led to the highest *p*_near_ early in the season (original SDP model). The low reproductive fitness function with the longest *L*_min_ (13.0 m) led to the highest *p*_off_ late in the season (adjusted length SDP model). Weekly habitat use patterns were approximately the same for the medium reproductive fitness function with *L*_min_ > 12.1 and for all high reproductive fitness function SDP models. Therefore, we chose one of those models (adjusted function SDP model: medium fitness function, *L*_min_ = 12.7) as a representative model. The two low reproductive fitness function models represented the most extreme *p*_near_ and *p*_off_ values.

**Fig. 4:**
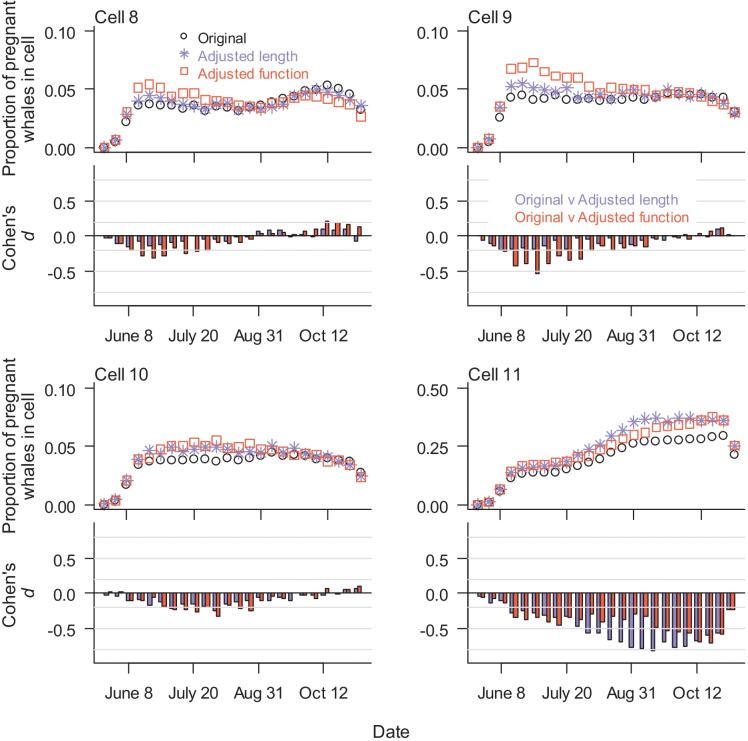
SDP model results of mean weekly proportion of pregnant females in the four offshore cells without disturbance. Three combinations of reproductive fitness function and minimum maternal length were used in analyses of the consequences of disturbance: Original = low fitness function, minimum length = 11.0 (black circles), adjusted length = low fitness function, minimum length = 13.0 (purple stars), and adjusted fitness = medium fitness function, minimum length = 12.7 (orange squares)

Comparing cell-use predictions between the three SDP models without disturbance, the original SDP model predicted a higher proportion of nearshore females, primarily in Cells 3 and 5 (|*d*| between 0.2 and 0.5). For all three models, use in offshore Cells 8, 9, and 10 was generally constant once whales arrived but declined in the last several weeks (Fig. 4). In contrast, the proportion of the population in Cell 11 increased throughout the season. The adjusted function model had a higher proportion of females in Cells 8 and 9 at the beginning of the season and Cell 10 in the middle of the season with |*d*| between 0.2 and 0.5 (Fig. 4). There was a moderate-to-high difference in the proportion of animals in Cell 11, with highest proportions for the adjusted length SDP model and lowest for the original SDP model (Fig. 4).

### Objective 2: Comparison of modeled habitat use and reproductive success without disturbance and with acoustic disturbance data from 2015

**Fig. 5:**
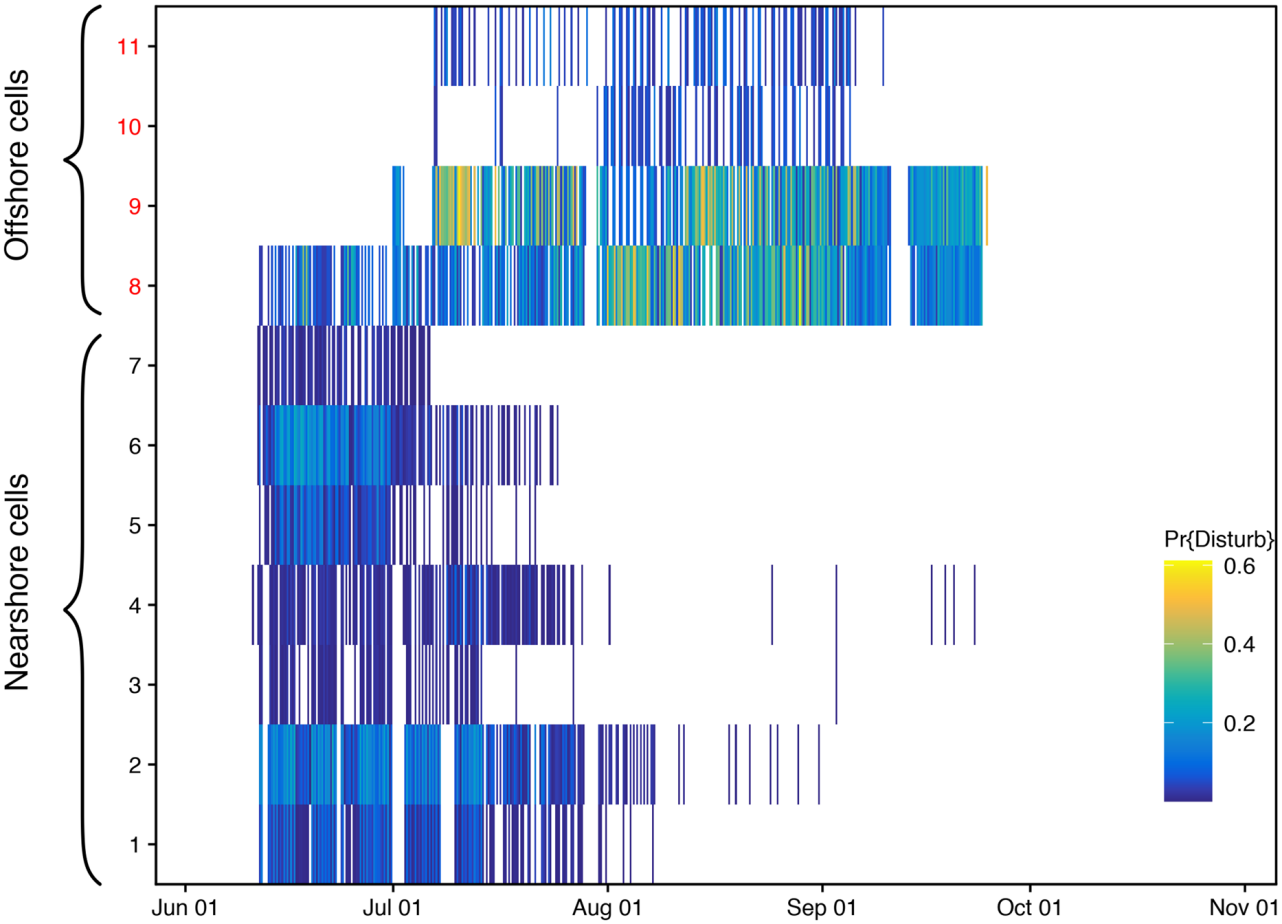
Disturbance probability by cell. Probability of disturbance is defined as the proportion of acoustic data points within a cell ≥ 163 dB re 1µPa^2^ SPL measured every 300 s, pooled in 6-h bins

The probability of disturbance in a 6-h period, i.e., the probability an individual could be exposed to air gun sound levels of 163 dB re 1µPa^2^ or more and move away to the closest undisturbed cell if it was in that cell, was spatially and temporally variable and reflects the seismic survey activities (Fig. 5). Disturbance in the nearshore cells occurred earlier in the season, and coastal nearshore area (Cells 1, 3, and 5) overall had lower disturbance probabilities than their equivalent non-coastal area (Cells 2, 4, and 6, respectively). Offshore Cell 8 had some level of air gun sound exposure for almost the entire seismic survey period. The probability of disturbance was highest in offshore Cells 8 and 9. SDP models predicted the proportion of disturbed pregnant females increased through the beginning of the season and peaked around 0.035 throughout August then sharply declined (Fig. S4).

**Fig. 6:**
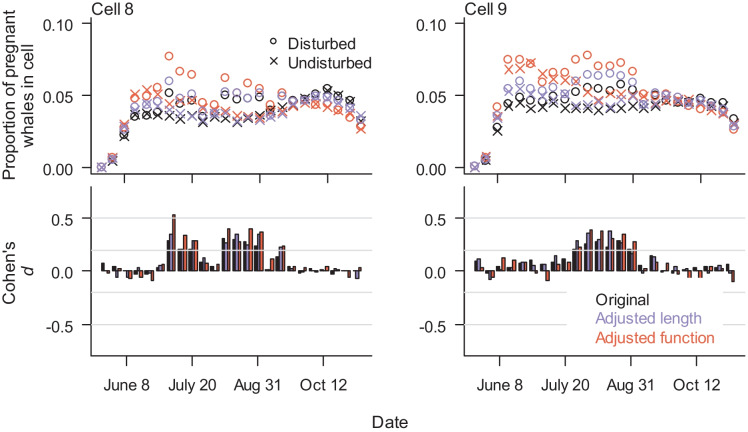
Model results of proportion of pregnant females in offshore Cells 8 and 9, binning 6-h periods into weeks, with and without disturbance (open circles and crosses, respectively). Cohen’s *d* values compare disturbed and undisturbed scenarios for three SDP models. Cohen’s *d* > 0 indicates higher proportions in a cell under disturbed conditions. Gray lines in Cohen’s *d* graphs indicate thresholds between no-to-little effect (|*d*|< 0.2), small (0.2 ≤|*d*|< 0.5), intermediate (0.5 ≤|*d*|< 0.8), and large effect (|*d*|≥ 0.8). Disturbance had no-to-little effect in proportions of pregnant females in all other cells

Using the 2015 measured acoustic values, forward simulations from the three representative models were used to predict the proportion of animals disturbed in 2015 and to compare cell use with and without disturbance. Although a higher proportion of pregnant females were disturbed in the adjusted function model, Cohen’s *d* values indicate no-to-little effect (|*d*|< 0.1) of SDP model choice on the predicted proportion of pregnant females disturbed (Fig. S4). Cohen’s *d* comparisons between the disturbed and undisturbed scenarios indicated there was little effect of disturbance on nearshore cell usage for all three SDP models (Cohen’s *d* between − 0.2 and 0.2). Regardless of SDP model type, disturbance had a no-to-small effect on use patterns in offshore Cells 10 and 11 and a small-to-medium effect on cell use in Cells 8 and 9, showing an increased proportion of females in cells with higher probability of disturbance (Fig. 6).

### Objective 3: Comparison of model results with observed gray whale distribution and photo-identification data from 2015

**Fig. 7:**
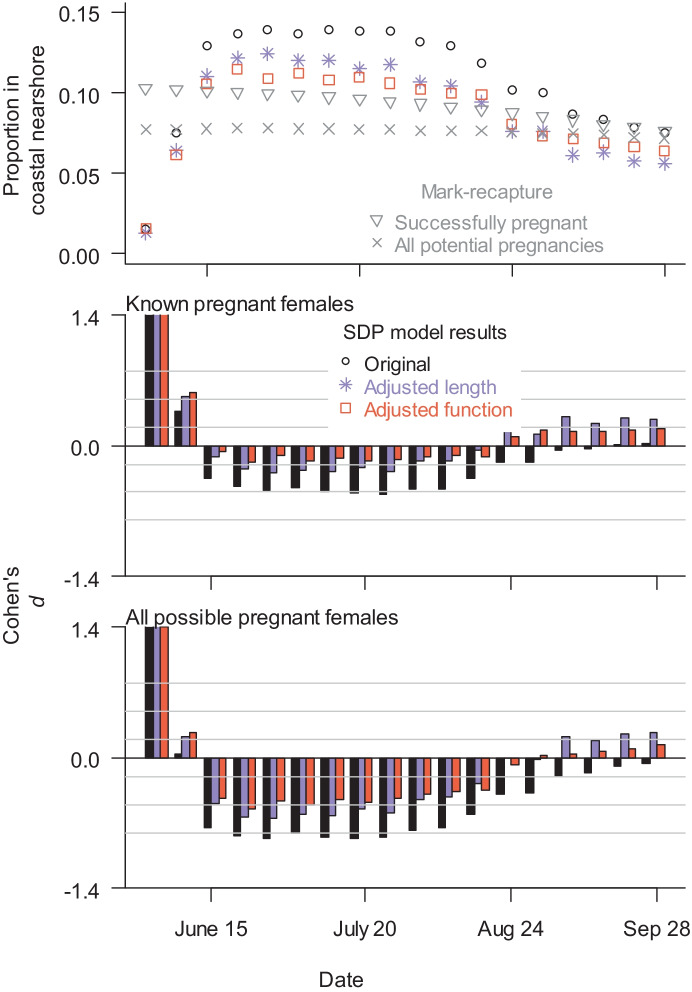
Mean proportion of pregnant females in coastal nearshore area (Cells 1, 3, 5, and 7), comparing predictions from three SDP models with regression estimates from photo-identification data (top). Cohen’s *d* values comparing SDP models with the proportion of known pregnant females nearshore (middle) and the proportion of all potentially pregnant females nearshore (bottom). Cohen’s *d* > 0 indicates higher estimates from empirical data, while Cohen’s *d* < 0 indicates higher predictions from SDP models. Gray lines in Cohen’s *d* graphs indicate thresholds between no-to-little effect (|*d*|< 0.2), small (0.2 ≤|*d*|< 0.5), intermediate (0.5 ≤|*d*|< 0.8), and large effect (|*d*|≥ 0.8)

Photo-identification surveys were conducted on 99 days from June 1 to October 1, 2015 (Supp Data) (Schwarz et al., 2022). Zero-inflated beta regression analysis was used to estimate the proportion of known pregnant and possibly pregnant females utilizing the coastal nearshore cells (*p*_preg_) over the foraging season suggested a slight decline in the proportion with time (Tables S2 and S3, Fig. 7). Proportions increased with better environmental conditions (lower Beaufort sea state and better visibility).

The number of scan surveys conducted in each cell ranged from 962 to 1281 (Table S4). The number of surveys with whale density > 0 ranged from 15 to 1019 depending on cell. Whale density was extremely low in Cell 2; of the 962 scan surveys that covered that cell, only 15 had a whale density > 0. Therefore, sample size limited the gamma regression analysis to one explanatory variable per model in Cell 2. Hurdle-gamma regression results of whale density by cell consistently indicated the probability of whale density > 0 increased with the proportion of the cell covered during the scan survey (Tables S5 and S6). However, given that whales are seen, the density estimate declines with higher cell coverage (Tables S5 and S6). The proportion of the nearshore group that was known to be pregnant declined slightly over the season (Tables S7 and S8; Fig S4). The proportion of all potentially pregnant females in the nearshore group exhibited a u-shaped function over the season, although uncertainty was high (Tables S7 and S8; Fig S4).

**Fig. 8:**
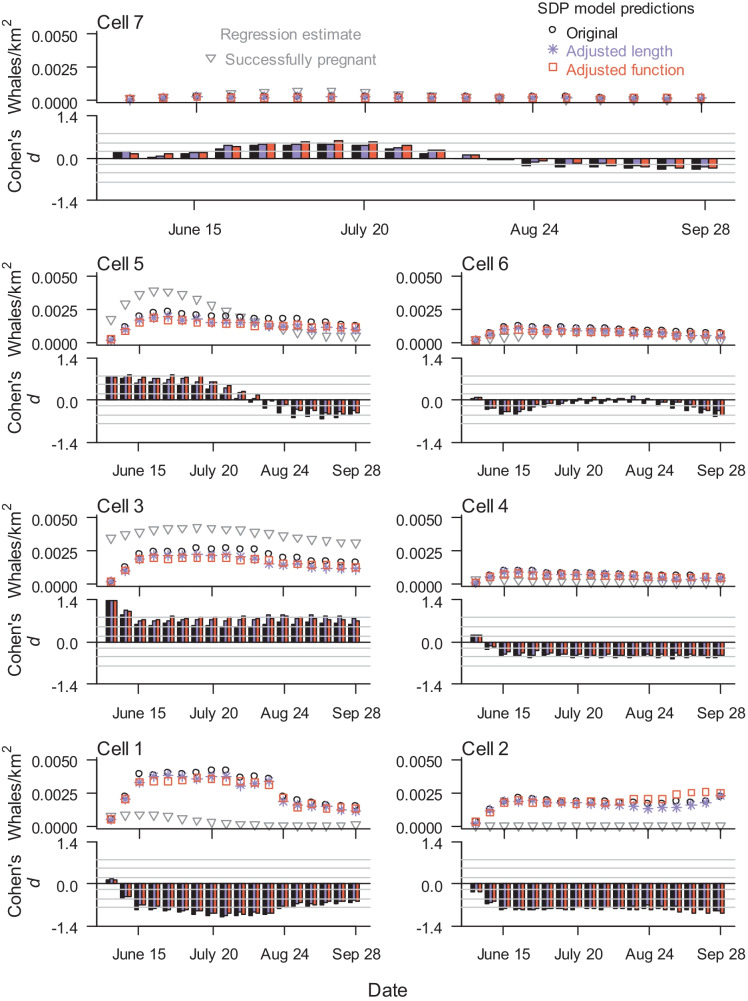
Density of known pregnant females in the nearshore area (Cells 1–7) over the foraging season, comparing SDP model predictions with regression estimates from empirical data, assuming ideal sighting conditions (visibility = 1, Beaufort = 0). Cohen’s *d* values > 0 indicate a higher regression estimate than SDP model predictions, while Cohen’s *d* values < 0 indicate a lower regression estimate than SDP model predictions. Gray lines in Cohen’s *d* graphs indicate thresholds between no-to-little effect (|*d*|< 0.2), small (0.2 ≤|*d*|< 0.5), intermediate (0.5 ≤|*d*|< 0.8), and large effect (|*d*|≥ 0.8). For density of all possibly pregnant females, see Figure S3

Regression estimates indicated the highest density of successfully pregnant females was in the coastal nearshore Cells 3 and 5 (Fig. 8). Density estimates were highest in the coastal nearshore area (Cells 1, 3, and 5) and lowest in Cells 4, 6, and 7.

**Fig. 9:**
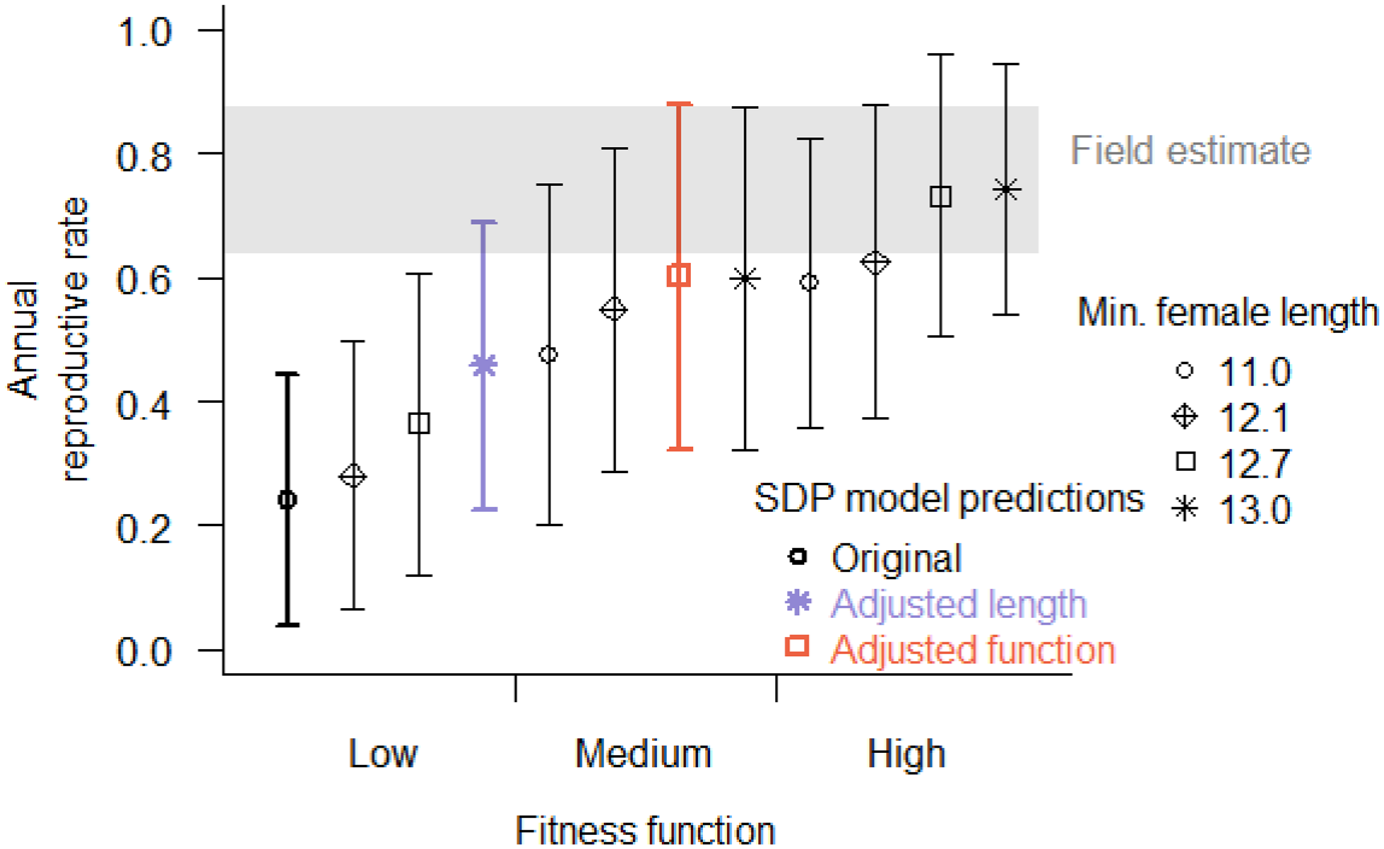
Predicted probability of successful reproduction from 12 SDP simulations with varying reproductive fitness functions and minimum maternal length compared to the possible range of observed reproductive rates in 2015 (gray-shaded area). The three SDP models used for habitat use comparisons are highlighted for reference. Points are means and whiskers are ± 2 sd

Fourteen new calves were identified in the Sakhalin feeding areas in 2016 (Tyurneva et al., 2018), and eleven calves were identified in 2015 (Yakovlev et al., 2016). With a minimum inter-birth interval of 2 years, eleven reproductive females would not be able to return with a calf in 2016. Our dataset included a total of 27 reproductive females based on their previous reproductive history. An additional six reproductive females have been identified by another group (Cooke et al., 2017), meaning we never had confirmed sightings of those individuals with a calf. Assuming 27 reproductive females in the population, only 16 females could be pregnant in 2015 since the other 11 produced a calf in 2015, leading to a measured successful reproductive rate of 0.88. If we include the additional six females in the total, the measured successful reproductive rate is 14/22 or 0.64 (Fig. 9).

Given the differences in predicted cell use, the three representative SDP models were used to compare SDP predictions with empirical estimates of *p*_near_, nearshore density, and reproduction. SDP predictions of *p*_preg_ in the coastal nearshore area (Cells 1, 3, 5, and 7) increased dramatically from June 1 through the first 4 weeks of the season followed by a gradual decline throughout the rest of the season (Fig. 7). Compared to other SDP models and empirically derived estimates, the original SDP model predicted the highest *p*_preg_ (Fig. 7). Empirical estimates under ideal conditions (Beaufort = 0 and visibility = 1) were higher than SDP model predictions at the beginning and end of the season and lower during the middle of the season (Fig. 7). Cohen’s *d* values indicate empirical estimates were much higher than SDP model predictions (large effect) during the first week.

All three SDP models predicted a higher whale density in Cells 1, 2, and 4 than the regression estimates, with large Cohen’s *d* effects. The SDP models predicted lower whale densities in Cell 3 compared to regression results, with intermediate-to-high Cohen’s *d* values. Once the females arrived, all three SDP models predicted a decline in the nearshore density of pregnant females as the foraging season progressed, a similar trend generally mirrored in the regression results. Regression estimates indicated the peak whale density and the rate of the subsequent decline were higher in Cells 5 and 7 than what was predicted from the SDP model. In particular, Cell 5 Cohen’s *d* values indicated an intermediate effect in the first part of the season (Fig. 8). Trends in density estimates of all possible pregnant females were similar to estimates for the smaller group of known pregnant females with the exception of Cell 3 where density of females increased at the end of the season (Fig. S5). The proportion of all possibly pregnant females within the photo-identified coastal nearshore area group of animals was a function of week^2^ (Fig. S6), which when combined with scan regression analyses created the U-shaped density estimates in Cell 3.

SDP predictions of the probability of successful reproduction increased with increased minimum maternal length (*L*_min_) and with changes in fitness function (Fig. 9). Mean SDP predictions of reproductive rate ranged from 0.24 to 0.74, with two means of the models falling within the range of empirical values (high reproductive fitness function with *L*_min_ > 12.1 m). Of the 12 SDP model simulations, the field reproductive rate range was included in the ± two standard deviation span around SDP predicted means for all but three models (low reproductive fitness function with *L*_min_ < 13.0 m). Cohen’s *d* values indicated no-to-little effect of disturbance on reproductive rate predictions (|*d*|< 0.2).

## Discussion

### Objective 1: Application of SDP model with different reproductive female lengths and fitness functions

Reproductive fitness functions are one of the greatest sources of uncertainty for SDP models, particularly for large whales, because there is little-to-no empirical data to determine their shape or limits. Therefore, it was important to explore various functions to determine their effect on habitat use, probability of disturbance, and probability of reproductive success. Overall, our modifications of the reproductive functions used in McHuron et al. (2021) did not result in large differences in nearshore-offshore habitat use patterns or proportion of animals disturbed. Predictions from all of the SDP models indicated a general decline over the season in nearshore habitat use that coincided with an increase in use of offshore Cell 11.

Increased use of the most prey energy-dense area (Cell 11) over the season aligned with the females’ higher energetic needs as the season progressed. When the pregnant female population consisted of smaller animals (*L*_min_ = 11.0 m), a lower proportion of females utilized Cell 11 because their energetic needs were lower. Future analysis of offshore whale density from line-transect vessel surveys could further our understanding of those links.

### Objective 2: Comparison of modeled habitat use and reproductive success without disturbance and with acoustic disturbance data from 2015

Using measured air gun sound levels as the only source of disturbance, SDP models predicted a moderate increase in use of offshore Cells 8 and 9, with small changes in other cells, including nearshore. In the SDP models, disturbed whales moved to the nearest undisturbed cell, and the SDP model Cells 8 and 9 were defined as each other’s closest cell. When disturbance was high in Cell 8, it was comparatively lower in Cell 9 and vice versa. The predicted pattern of higher proportions in Cells 8 and 9 during disturbance indicates movement between the cells. While the model indicated offshore females may have lost foraging time when moving out of a disturbed cell, they moved to cells with equal or more prey availability, effectively compensating for lost energy later in the season.

However, the assumption that whales would move to the closest undisturbed cell was not based on empirical data, since the magnitude of the behavioral response (how far an animal moves and how much foraging is lost) has yet to be quantified. Whales could show displacement farther from disturbed cells, which could lead to different predicted habitat use patterns and energetic outcomes. While disturbance had the most impact on use of Cells 8 and 9 (which have lower prey energy than Cell 11), the location of the highest offshore prey resources may not be consistent (Blanchard pers. com.). Thus, future seismic surveys with similar spatial and temporal patterns may not have the same impact on whale foraging or distribution.

### Objective 3: Comparison of model results with observed gray whale distribution and photo-identification data from 2015

SDP model predictions of the proportion of pregnant females utilizing the coastal nearshore area in 2015 were similar to estimates from photo-identification data. However, the data indicated the animals were present in the area before data collection began. Although arrival time of gray whales to Sakhalin Island is unknown, very few animals are successfully identified at the beginning of the season, and discovery rates were relatively constant throughout 2015 (Schwarz et al., 2022). That information led us to believe that whales were still arriving after photo-identification data collection began. Therefore, we assumed an arrival time of June 15 ± 5 days in the SDP models but did not account for demographic-specific differences in arrival time. For example, the identified pregnant and potentially pregnant females in 2015 were first seen on June 2nd and June 7th, respectively. The first calf was identified on June 24th, and the first mother was identified on July 7th, indicating a much earlier arrival time for pregnant females. Model sensitivity analysis from the initial SDP effort indicated arrival date had no effect on successful reproduction or maternal survival (McHuron et al., 2021) nor on the timing of the shift between nearshore and offshore cells. So, a change in the SDP arrival date would only change whale proportions in the nearshore and offshore area during the first few weeks of the season. Studies to determine arrival times earlier than June 1st would be limited due to ice and fog, but future model assumptions about arrival time should be developed with demographic-specific information in mind.

Within the nearshore area, SDP models predicted a higher density of animals in Cells 1, 2 (large effect, |*d*|≥ 0.8), and 4 (small-to-intermediate effect, 0.2 ≤|*d*|< 0.8) and a lower density in Cell 3 (intermediate-to-large effect, 0.5 ≤|*d*|) compared to density estimates from scan surveys. In fact, density estimates from scan surveys indicated an almost complete lack of whales in the southeastern part of the nearshore foraging area (Cell 2). Inter-annual whale densities in the southern part of the nearshore area (Cells 1 and 2) have been highly variable since density data have been collected (Exxon Neftegas, 2018), with Cell 1 usually an area of lower prey biomass (Blanchard et al., 2019, 2022a, b). Little is known about whale prey energy in Cell 2. Amphipods are considered the whales’ prey of choice (Budnikova & Blokhin, 2012; Dunham & Duffus, 2001; Oliver et al., 1983), and we adjusted habitat use probabilities in the SDP model as a function of the proportion of energy from amphipods. We also assumed in SDP models that Cell 2 prey energy had the same mean prey energy value as other offshore cells (Cells 4 and 6) with lower variability (McHuron et al., 2021). Clearly the adjustment and assumption relative to prey availability need to be reassessed.

Cell 3 includes the mouth of Piltun Bay, and more-detailed analysis of the distribution data indicated whale density in that area was particularly high (Gailey et al., 2022b). Empirical density estimates in Cell 3 may be a reflection of high densities of mother-calf pairs and young animals concentrated at the mouth of Piltun Bay. The highest observed densities were within 1 km of shore (Gailey et al., 2022b), while pregnant females are more likely to be seen further from shore (Sychenko, 2011). Thus, our adjustment to determine the density of pregnant females from the density of all whales may not be accurate for this area since non-pregnant females may make up a higher proportion of animals in the area.

Conversely, if pregnant females are foraging < 1 km from shore, benthic prey data (a key input in the SDP model) may not be an accurate representation of the level of prey energy available to pregnant whales in Cell 3 because nearshore whales are regularly seen foraging in depths considered too shallow to safely or adequately sample benthic prey (< 7 m) (Blanchard et al., 2022b).

Density estimates from scan data indicated highest use of Cell 7 from late June through early July, which was not predicted using the SDP model. Analysis of smaller 1-km^2^ blocks indicated a higher density of gray whales farther than usual from shore in Cell 7 (Gailey et al., 2022b) which could have been related to a seasonal patch of high energy sand lance (*Ammodytes* sp.) (Blanchard et al., 2022a). Gray whales have previously been observed in that area feeding on sand lance (Fadeev, 2011). Because of the limited structure of the distribution of prey energy across space and time, the SDP models were not sensitive enough to capture behavioral differences driven by seasonal prey patches. That is, limitations in temporal and spatial benthic biomass sampling (Blanchard et al., 2022b) restricted us to one overall prey energy distribution for the entire foraging season in Cell 7 (McHuron et al., 2021; Maresh et al., 2022).

While disturbance had no-to-little effect on predictions of reproductive rate from SDP models, predictions were generally lower than the range of reproductive rate observed in 2015. However, of the 12 SDP model simulations that included 2015 disturbance, only three models (low reproductive fitness function and minimum length < 13.0) were inconsistent with observations. Analysis of long-term photo-identification data of Sakhalin Island gray whales estimated age at sexual maturity at 9.0 years old (7.7–11.2 years old) (Bradford et al., 2010; Cooke, 2010), which is consistent with findings in eastern gray whales (Rice & Wolman, 1971). An additional 10.5-year-old female was first seen with a calf in 2014 (Schwarz et al., 2022). Based on gray whale growth rates (Agbayani et al., 2020), we estimated roughly 50% of females would have successfully reproduced by age 9 years (with a mean value of 12.1 m in length) using both the medium and high reproductive fitness functions (Fig. S3). Thus, both the empirical probability of reproductive success and age/size at sexual maturity support use of the medium and high reproductive fitness functions. Empirically determined age at sexual maturity estimates a minimum length around 11.7 m (mean length at 7.7 years old) and does not support a minimum maternal length > 12.1 m, which would translate to a minimum age of sexual maturity > 10 years old.

The empirical probabilities of successful reproduction would be biased high if the counted number of pregnant females was low. The long-term dataset (2002–2015) shows that the mothers are unknown for 38% of identified calves, likely due to variability in timing and photo-identification data collection effort each year, as calves could have separated from their mothers prior to data collection. Sakhalin Island gray whales have also been sighted off Kamchatka Peninsula, including mother-calf pairs that have separated before arrival at Sakhalin Island (Tyurneva et al., 2013). Therefore, it is likely that some Sakhalin Island reproductive females have not yet been identified. In 2015, 11 individuals of reproductive age (> 6 years old) were either female or of unknown gender and could have been unidentified first-time mothers in 2016. An additional 81 identified whales were of unknown reproductive status (two female, 79 unknown gender). Four of those individuals were first identified in 2015. Based on sighting histories, the other 77 animals had a mean minimum age of 11 years, with 71% of them (*N* = 55) at least 10 years old, at the higher end of the range for age of sexual maturity (Cooke, 2010; Rice & Wolman, 1971). Overall, several attributes of the photo-identification data lead to the potential for underestimates of the number of pregnant females in 2015.

If SDP models were adjusted to more-closely simulate the habitat use patterns seen from empirical data (no-to-little use of Cells 1 and 2, higher use of Cell 3), SDP predictions of reproductive rates would most likely remain the same, either with or without disturbance. McHuron et al. (2021) tested several theoretical disturbance scenarios that involved high levels of disturbance in all nearshore cells, resulting in no effect on predictions of successful reproduction. Alternatively, given that prey energy in Cell 11 can be up to ten times higher than energy in nearshore cells, exclusion from offshore cells could result in complete reproductive failure and maternal death (McHuron et al., 2021).

## Conclusion

To identify the behavioral choices and demographic results of disturbance on pregnant western gray whales during one foraging season, a stochastic dynamic programming (SDP) model has been developed (McHuron et al., 2021). However, important underlying model assumptions about minimum maternal length (*L*_min_) and the reproductive fitness functions (*R*_fit_) remained untested. Coincidentally, multiple seismic surveys were conducted in 2015 off the northeast coast of Sakhalin Island, Russia, in and near an important foraging habitat, which could lead to decreased gray whale foraging activity and reduced population growth via lower survival or reproduction. We varied assumptions about *L*_min_ and *R*_fit_ to determine their effects on SDP predictions of habitat use, proportion of animals disturbed (*p*_dist_), and reproductive success (*ϕ*), as well as the effects of disturbance from air gun sounds on predictions of habitat use and *ϕ*.

Benthic prey surveys, photo-identification studies, and distribution sampling occurred in conjunction with seismic surveys in 2015, providing a unique opportunity to compare output from SDP models with empirical observations of whale distribution, behavior, and vital rates. Resulting SDP predictions indicated higher nearshore habitat use with shorter *L*_min_, which reflects the expected differences in behavior based on size-specific energetic needs. Changes in *L*_min_ and *R*_fit_ had no-to-little effect on predictions of *p*_dist_ or *ϕ*. Disturbance had a small-to-medium effect on predicted cell use in the northern offshore foraging area where disturbance was highest, showing an increased proportion of females in cells with higher probability of disturbance. SDP predictions of large-scale habitat use over the season were generally similar to values and trends seen from photo-identification and distribution data. However, empirical estimates of the proportion of pregnant females nearshore were much higher than SDP model predictions (large effect: |Cohen’s *d*|> 0.8) during the first week, so arrival date was most likely too late in the SDP model. Within their nearshore habitat, the SDP model overestimated whale density in the south and underestimated density around the mouth of Piltun Bay compared to empirical estimates. SDP predictions of *ϕ* increased with increased *L*_min_ and *R*_fit_, and measured *ϕ* was a possible outcome in nine of the 12 SDP models.

Overall, this work, using data from a specific well-studied situation, underscores the potential usefulness of SDP modeling as a means of quantitatively applying the PCOD framework. However, and not surprisingly, SDP model predictive abilities can be limited due to assumptions in the model and parameter inputs. Sensitivity analyses can test the effects of SDP model assumptions on predictions and provide valuable information about where researchers need to focus data collection to improve SDP model accuracy. For example, measured lengths of known-age animals, particularly reproductive females, would aid in predicting reproductive success. Comparisons with field studies also identify SDP model limitations. For example, the SDP model could not capture some detailed gray whale nearshore spatial distribution because the temporal and spatial scales of prey input parameters (derived from data) were too coarse. Notably, the assumptions and limitations in the SDP model did not affect the comparisons between disturbed and undisturbed scenarios. If future SDP models are expanded to determine the effects of disturbance over longer periods, accuracy of vital rates may become more important, and additional and expanded field studies will be needed to improve SDP model inputs and functions and validate SDP model outputs.

## Supplementary information

Below is the link to the electronic supplementary material.

Supplementary file1 (PDF 267 KB)
