# Supplementary material for: Gray whale habitat use and reproductive success during seismic surveys near their feeding grounds: comparing state-dependent life history models and field data
Source: Environ Monit Assess. 2022 Oct 18;194(Suppl 1):733. doi: 10.1007/s10661-022-10024-9 (PMC9579109; doi:10.1007/s10661-022-10024-9)
Supplement: Supplementary file 1 — Supplementary file1 (PDF 267 KB) [file 10661_2022_10024_MOESM1_ESM.pdf]

Variations in R_{fit}

The reproductive fitness function (R_{fit}) was calculated as the product of two functions:

$$R_{fit}(F|L_{mother}, L_{fetus}) = r(F|L_{mother}) \cdot r(L_{fetus})$$

Where $r(F|L_{mother})$ is the probability of successful reproduction as a function of maternal fat mass when the female stops foraging (F) for a given maternal length (L_{mother}), and $r(L_{fetus})$ is the probability of successful reproduction as a function of fetal length when the female stops foraging (L_{fetus}). We investigated changes in foraging location and annual reproductive success for three R_{fit} functions using different combinations of two $r(F|L_{mother})$ functions and three $r(L_{fetus})$ functions.

Villegas-Amtmann et al. (2015) calculated $r(F|L_{mother})$ using a distribution of maternal lengths and assumed a female utilized stored energy for her own metabolic needs before provisioning her fetus or newborn calf. McHuron et al. (2021) used the same $r(F|L_{mother})$ calculations but used a maximum fat mass (F_{max_2021}) that was 89-94% of maximum fat mass estimates in Villegas-Amtmann et al. (2015) (F_{max_2015}, Table S1). We increased $r(F|L_{mother})$ by shifting the reproductive function over by the difference in maximum fat mass estimates.

$$r_{new}(F|L_{mother}) = r_{old}(F + (F_{\max\_2015} - F_{\max\_2021})|L_{mother})$$

The shifted method essentially reduced the amount of fat needed to successfully reproduce by reducing over-winter female metabolic needs, reproductive costs, or a combination of both. The mean increase in $r(F|L_{mother})$ ranged from 0.008 to 0.081 depending on maternal length (Table S1). For maternal lengths greater than 11.5 m, the shifted reproductive success for a given mass and length was roughly the same as the original reproductive success for a given mass and length one meter shorter: $r_{new}(F|L_{mother}) = r_{old}(F|(L_{mother} - 1))$ (Fig. S1).

We used a sigmoid function for $r(L_{fetus})$ with two variables: the fetal length at which the probability of reproductive success is 0.5 (l_{50}) and an exponent value (a) that describes the rate at which reproductive success changes before and after l_{50}.

$$r(L_{fetus}) = \frac{L_{fetus}^{a}}{L_{fetus}^{a} + l_{50}^{a}}$$

A higher value of a indicates a higher rate of change with fetal length. In McHuron et al. (2021) $l_{50} = 1.96$ and $a = 3$ ($r_1(L_{fetus})$). The values of l_{50} and a are unknown, so we created an additional function by changing l_{50} to 2.16 $r_2(L_{fetus})$. A third function changing both parameters: $l_{50} = 2.16$ and $a = 6$ ($r_3(L_{fetus})$, Fig. S2).

Based on the value of R_{fit} for longer fetuses, we defined the three R_{fit} functions as

Low: $R_{fit}(F|L_{mother}, L_{fetus}) = r_{old}(F) \cdot r_1(L_{fetus})$ (McHuron et al. 2021),

Medium: $R_{fit}(F|L_{mother}, L_{fetus}) = r_{new}(F) \cdot r_2(L_{fetus})$, and

High: $R_{fit}(F|L_{mother}, L_{fetus}) = r_{new}(F) \cdot r_3(L_{fetus})$

Table S1. Maximum maternal fat mass by length estimated using two different methods and the mean change in the probability of reproduction as a function of fat mass by length after shifting the function.

Maternal length (m)	Maximum fat mass (kg x 10^3) Villegas-Amtmann et al. (2015)	McHuron et al. (2021)	Mean change in $r(F \mid L_{mother})$
11.0	7.6	7.2	0.008
11.1	7.9	7.4	0.011
11.2	8.2	7.6	0.015
11.3	8.5	7.8	0.018
11.4	8.7	8.0	0.022
11.5	9.0	8.2	0.027
11.6	9.3	8.4	0.031
11.7	9.6	8.6	0.036
11.8	9.8	8.8	0.041
11.9	10.1	9.1	0.045
12.0	10.4	9.3	0.050
12.1	10.7	9.5	0.054
12.2	11.0	9.8	0.059
12.3	11.2	10.0	0.063
12.4	11.5	10.3	0.066
12.5	11.8	10.5	0.070
12.6	12.1	10.8	0.072
12.7	12.3	11.0	0.075
12.8	12.6	11.3	0.076
12.9	12.9	11.6	0.078
13.0	13.2	11.8	0.079
13.1	13.4	12.1	0.081
13.2	13.7	12.4	0.081
13.3	14.0	12.7	0.080
13.4	14.3	13.0	0.080
13.5	14.5	13.3	0.079
13.6	14.8	13.5	0.078
13.7	15.1	13.9	0.076
13.8	15.4	14.2	0.073
13.9	15.6	14.5	0.071
14.0	15.9	14.8	0.068

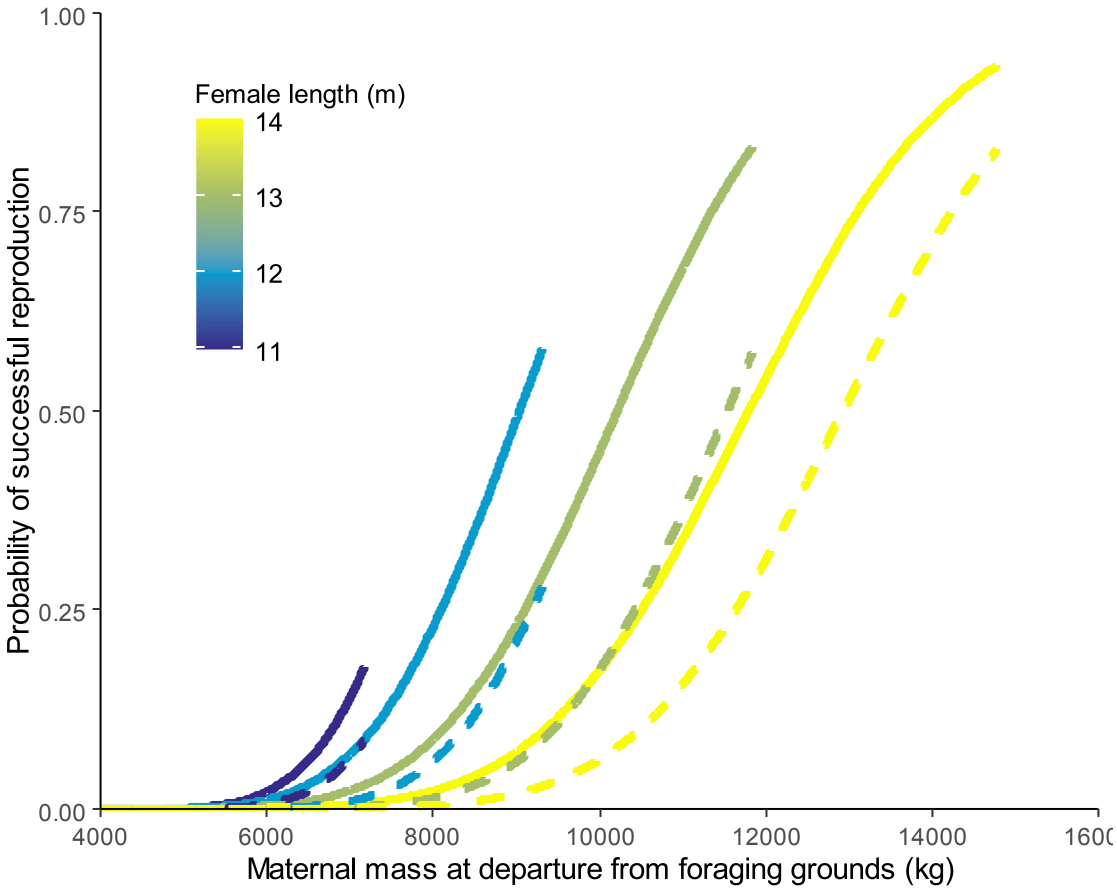

Fig. S1. The probability of successful reproduction as a function of maternal mass at the time of departure from the foraging grounds given four maternal lengths ($r(F|L_{mother})$). Dashed lines are values for the original function and solid lines are values for the shifted function. Graph shows selected female lengths of 11, 12, 13, and 14 m.

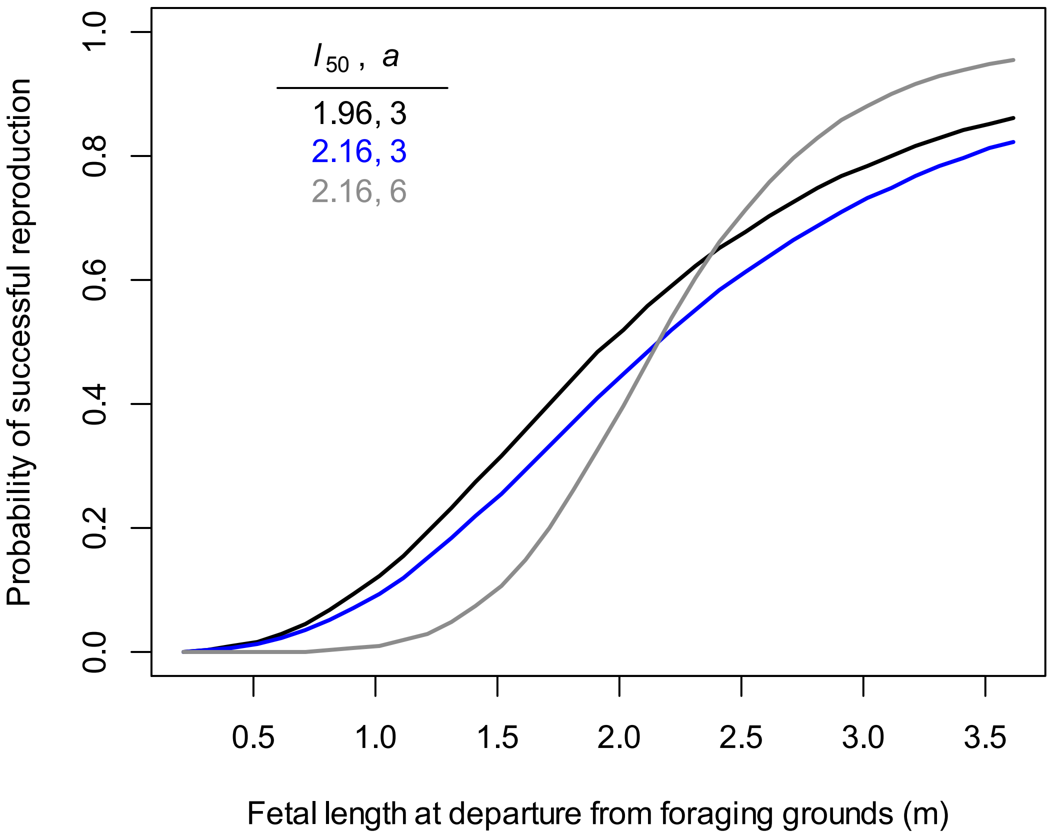

Fig. S2. Probability of successful reproduction as a function of fetal length at the time of departure from the foraging grounds ($r(L_{fetus})$), where $r(L_{fetus}) = \frac{L_{fetus}^a}{L_{fetus}^a + l_{50}^a}$. The three combinations of values for l_{50} and a represent $r(L_{fetus})$ for the low, medium, and high R_{fit} functions, respectively.

Determination of minimum limit on reproductive female length

SDP model results indicate female length affects reproduction, with smaller females less likely to successfully reproduce. In addition, reproductive females are not identified in the field until they have successfully reproduced (identified with a calf on the foraging grounds). Because the length distribution of successfully reproductive females is likely longer than what was originally sampled for the SDP model, we sampled from the original length distribution as well as three additional distributions. The original length distribution from McHuron et al. (2021) was normal with a mean of 12.7 m and 0.6 standard deviation, limited to a range between 11 and 14 m. The additional length distributions had the same normal mean and standard deviation but limited the minimum reproductive lengths to 12.1, 12.7, or 13.0 m. The lower limits were determined by estimating the length at which 50% of the females had successfully reproduced. To estimate the proportion of females that had successfully reproduced as a function of length, we combined the mean estimate of annual reproductive rate by length for the three fitness functions (Fig. 2) with mean female length at age, determined by Agbayani et al. (2020):

$$L_{female} = 13.06\left(1 - e^{-0.18(t+5.09)}\right)$$

Where L_{female} = female length in meters, and t = age in years. For both the medium and high reproductive functions, roughly 50% of females had successfully reproduced by 12.1 m long (Fig. S3). By 12.7 m long, 50% of all females under the original, low reproductive function had reproduced successfully, while almost all females had reproduced successfully using the medium and high reproductive functions. At 13.0 m long, all females had successfully reproduced for all three reproductive functions.

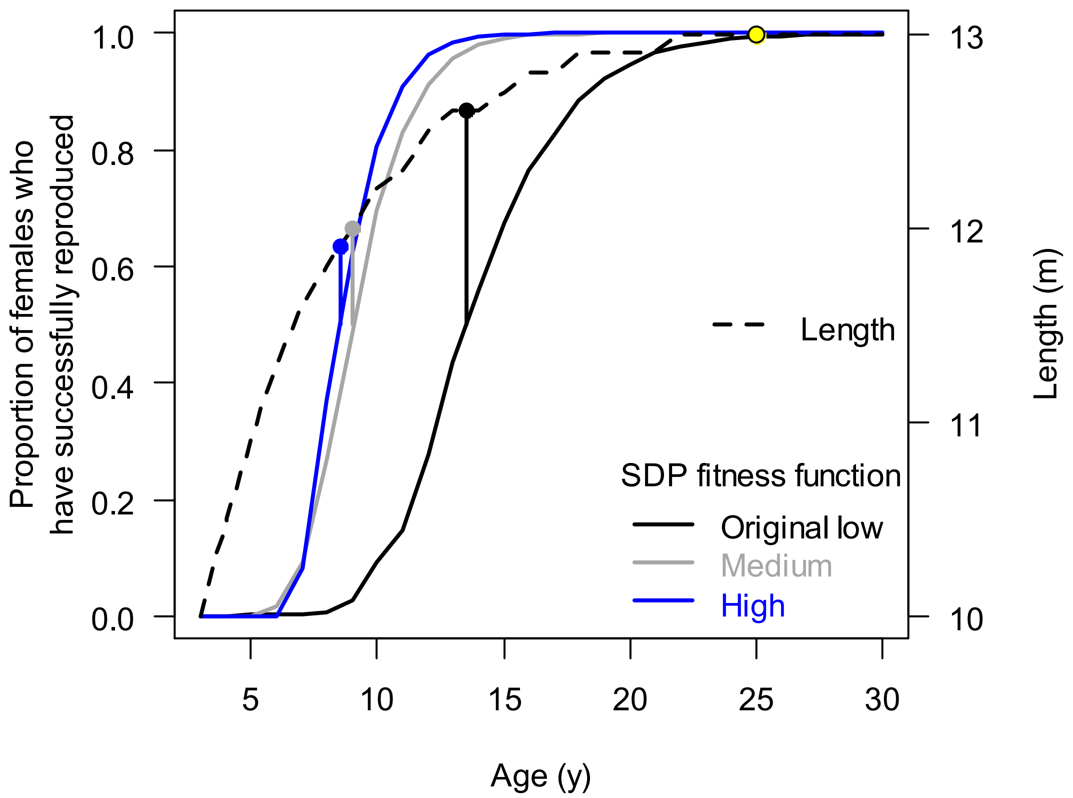

Fig. S3. Length at age and the proportion of females who have successfully reproduced by age, based on three reproductive fitness functions: original (low), medium, and high reproduction by length. Points indicate lengths at which 50% of females have successfully reproduced. Yellow dot is the length at which all females have reproduced for all three reproductive fitness functions.

References

Agbayani, S., Fortune, S. M. E., & Trites, A. W. (2020). Growth and development of North Pacific gray whales (*Eschrichtius robustus*). *Journal of Mammalogy, 100*(3), 742 - 754.

McHuron, E. A., Aerts, L., Gailey, G., Sychenko, O., Costa, D. P., Mangel, M., et al. (2021). Predicting the population consequences of acoustic disturbance, with application to an endangered gray whale population. *Ecological Applications*.

Villegas-Amtmann, S., Schwarz, L. K., Sumich, J. L., & Costa, D. P. (2015). A bioenergetics model to evaluate demographic consequences of disturbance in marine mammals applied to gray whales. *Ecosphere, 6*(10), 183, doi:10.1890/ES15-00146.1.

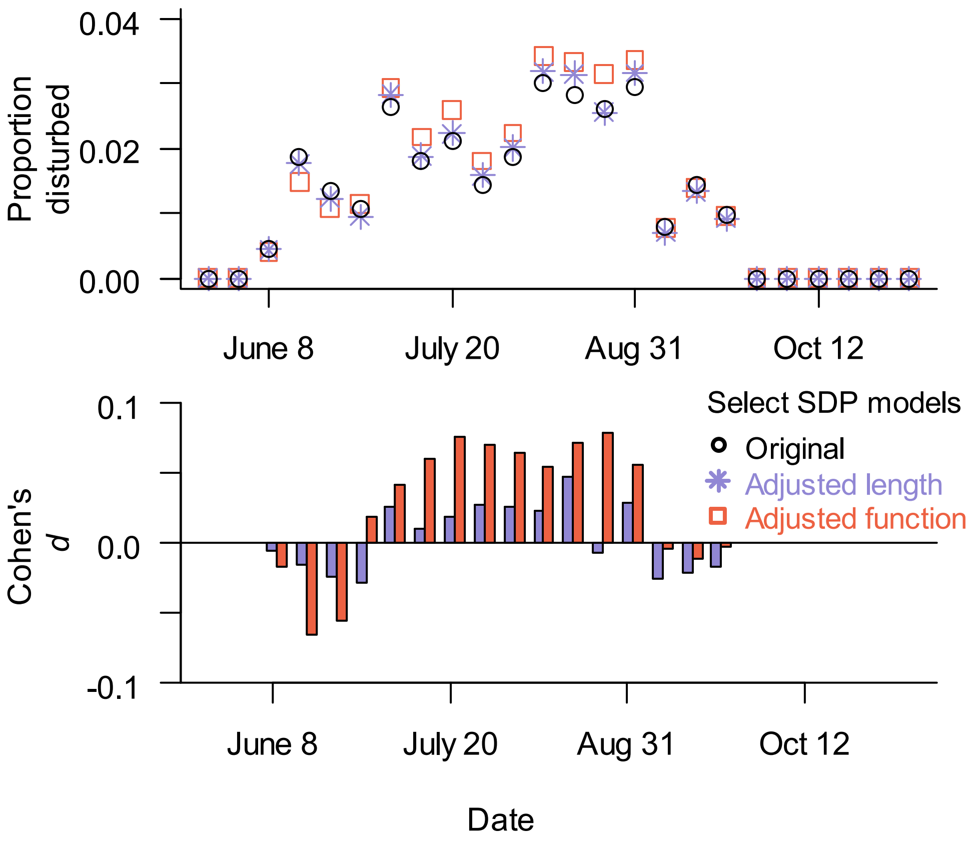

Fig. S4. Weekly mean proportion of the pregnant female population disturbed in six-hour bins across the 2015 foraging season for three SDP models (top). Cohen's *d* comparing the proportion disturbed from the Original SDP model with the Adjusted length and Adjusted function SDP models. In a comparison of Model A vs Model B, a positive Cohen's *d* value indicates a higher proportion of animals using Model A. Cohen's *d* values indicate no-to-little difference between the models ($|d| < 0.2$).

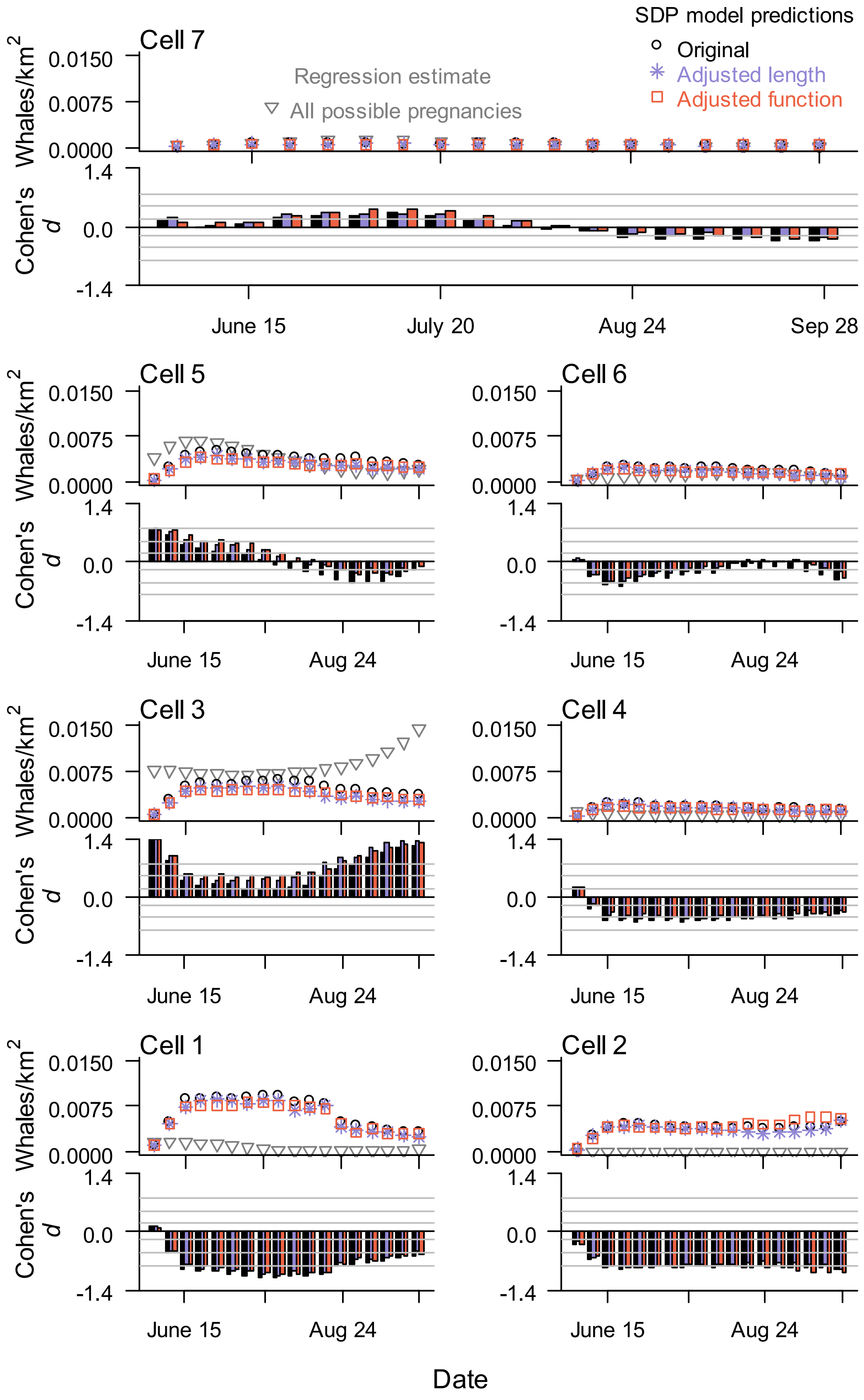

SDP model predictions
Original
Adjusted length
Adjusted function
Regression estimate
All possible pregnancies
Cell 7
Cell 5
Cell 6
Cell 3
Cell 4
Cell 1
Cell 2
Whales/km2
Cohen's d
0.0150
0.0075
0.0000
1.4
0.0
-1.4
June 15
July 20
Aug 24
Sep 28
Date

Fig. S5. Density of all possibly pregnant females in the nearshore area (Cells 1 - 7) over the foraging season, comparing SDP model predictions with regression estimates from empirical data, assuming ideal sighting conditions (visibility = 1, Beaufort = 0). Cohen's *d* values > 0 indicate a higher regression estimate than SDP model predictions, while Cohen's *d* values < 0 indicate a lower regression estimate than SDP model predictions. Gray lines in Cohen's *d* graphs indicate thresholds between no-to-little effect ($|d| < 0.2$), small ($0.2 \leq |d| < 0.5$), intermediate ($0.5 \leq |d| < 0.8$), and large effect ($|d| \geq 0.8$). For density of known pregnant females, see Figure 8.

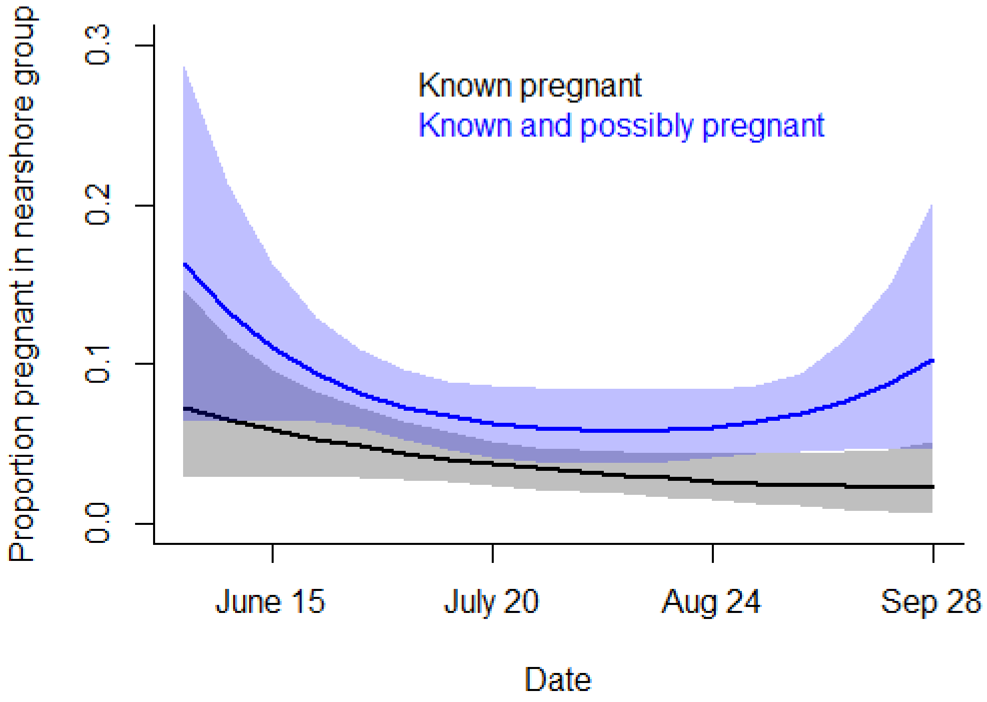

Fig S6. Proportion of the nearshore group that is pregnant as a function of time. Pregnant is defined as either all potential pregnancies (blue) or limited to only known pregnancies (black). Results of model stacking on week of year (Tables S6 and S7) with means as solid lines and shaded 95% posterior intervals.

Table S2. Bayesian model weights for estimating the proportion of pregnant females (p_{near}) in coastal nearshore cells (Cells 1, 3, 5, and 7) via zero-inflated beta regression. Likelihood is

$$bi_0(p_{near}|\alpha_B, \mu_B, \varphi) = \begin{cases} 1 - \alpha_B & \text{if } p_{near} = 0 \\ \alpha_B f(p_{near}|\mu_B, \varphi) & \text{if } p_{near} \in (0,1) \end{cases}, \text{ where}$$

$$f(p_{near}|\mu_B, \varphi) \sim Beta(\mu_B, \varphi) \text{ and } S_{y_p \neq 0} \sim Bin(S, \alpha_B)$$

The probability of seeing at least one pregnant female (α_B) and the mean of the beta distribution (μ_B) were independently estimated as functions of visibility, Beaufort sea state, week, and week^2. Week and week^2 were orthogonally transformed to avoid correlation between variables. The beta distribution precision parameter (φ) was assumed constant.

	Explanatory variables				Model weights			
					Known pregnancies		All possible pregnancies	
Model #	Visibility	Beaufort	Week	Week^2	α_B	μ_B	α_B	μ_B
1					0.000	0.407	0.000	0.003
2	x				0.000	0.592	0.225	0.568
3		x			0.000	0.000	0.210	0.188
4			x		0.221	0.000	0.000	0.211
5			x	x	0.000	0.000	0.000	0.000
6	x	x			0.304	0.000	0.001	0.000
7	x		x		0.000	0.001	0.000	0.000
8	x		x	x	0.113	0.000	0.130	0.000
9		x	x		0.362	0.000	0.272	0.031
10		x	x	x	0.000	0.000	0.000	0.000
11	x	x	x		0.000	0.000	0.162	0.000
12	x	x	x	x	0.000	0.000	0.000	0.000

Table S3. Posterior distribution of parameters (β) estimating the proportion of pregnant females in coastal nearshore cells (Cells 1, 3, 5, and 7) via zero-inflated beta regression. The probability of seeing at least one pregnant female (α_B) and the mean of the beta distribution (μ_B) were independently estimated as functions of visibility (V), Beaufort sea state (B), week of year (W), and week of year squared ($W2$). The beta distribution precision parameter (φ) was assumed constant. Means with standard deviations in parentheses. Blanks indicate a variable was not included in the final model average.

	Known pregnancies			All possible pregnancies		
Parameter	logit(α_B)	logit(μ_B)	log(φ)	logit(α_B)	logit(μ_B)	log(φ)
β_0	0.78 (1.26)	-1.657 (0.192)	4.744 (0.259)	1.82 (1.06)	-2.156 (0.236)	4.290 (0.207)
β_V	-0.20 (0.33)	-0.074 (0.074)		-0.27 (0.35)	-0.084 (0.094)	
β_B	-0.44 (0.44)			-0.46 (0.43)	-0.028 (0.069)	
β_W	-2.62 (2.60)			-1.74 (2.26)	0.087 (0.323)	
β_{W2}	-0.38 (1.38)			-0.33 (1.22)		

Table S4. Scan survey sample size by cell and cell coverage per scan (mean and standard deviation).

Cell	Total surveys	Total surveys with density > 0	Proportion of cell covered
1	962	198	0.76 (0.11)
2	962	15	0.67 (0.14)
3	1281	1019	0.66 (0.32)
4	1281	200	0.38 (0.21)
5	1182	870	0.80 (0.27)
6	1182	479	0.72 (0.27)
7	1057	334	0.60 (0.21)

Table S5. Bayesian model weights for estimating the density of gray whales (D) in each cell via hurdle gamma regression. Likelihood is

$$Gamma_0(D|\alpha_G, \mu_G, \omega) = \begin{cases} 1-\alpha_G & \text{if } D = 0 \\ \alpha_G f(D|\mu_G, \omega) & \text{if } D > 0 \end{cases}, \text{ where}$$

$$f(D|\mu_G, \omega) \sim Gamma(\mu_G, \omega) \text{ and } R_{D \neq 0} \sim Bin(R, \alpha_G)$$

The probability that D is greater than zero (α_G) and the mean of the gamma distribution (μ_G) were independently estimated as functions of the proportion of the cell covered during a scan survey, week, week^2, and week^3. Temporal variables were orthogonally transformed to avoid correlation between variables. The gamma distribution precision parameter (ω) was assumed constant. Sample size limited the number of explanatory variables to one per model for the Cell 2 gamma distribution.

	Explanatory variables				Model weights													
					Cell 1		Cell 2		Cell 3		Cell 4		Cell 5		Cell 6		Cell 7	
Model #	Coverage	Week	Week^2	Week^3	α_G	μ_G	α_G	μ_G	α_G	μ_G	α_G	μ_G	α_G	μ_G	α_G	μ_G	α_G	μ_G
1					0.043	0.000	0.509	0.000	0.000	0.000	0.000	0.117	0.000	0.000	0.003	0.040	0.007	0.050
2	x				0.000	0.016	0.491	0.843	0.172	0.000	0.534	0.000	0.124	0.031	0.010	0.000	0.099	0.000
3		x			0.000	0.057	0.000	0.157	0.000	0.079	0.000	0.022	0.000	0.001	0.000	0.012	0.001	0.000
4		x	x		0.000	0.000	0.000		0.000	0.344	0.003	0.000	0.000	0.001	0.000	0.116	0.007	0.000
5		x	x	x	0.179	0.091	0.000		0.000	0.577	0.000	0.000	0.124	0.275	0.000	0.004	0.000	0.375
6	x	x			0.001	0.000	0.000	NA	0.315	0.000	0.001	0.434	0.000	0.001	0.000	0.086	0.038	0.000
7	x	x	x		0.001	0.000	0.000		0.000	0.000	0.460	0.426	0.124	0.153	0.075	0.000	0.848	0.000
8	x	x	x	x	0.777	0.836	0.000		0.513	0.000	0.002	0.000	0.627	0.539	0.913	0.144	0.000	0.575

Table S6. Posterior distribution of parameters (β) estimating the gray whale density in each nearshore cell via hurdle gamma regression. The probability that gray whale density is greater than zero (α_G) and the mean of the gamma distribution (μ_G) were independently estimated as functions of the proportion of the cell covered during a scan survey (C), week (W), week^2 ($W2$), and week^3 ($W3$). Temporal variables were orthogonally transformed to avoid correlation between variables. The gamma distribution shape parameter (ω) was assumed constant. Means with standard deviations in parentheses. Blanks indicate a variable was not included in the final model average.

Cell	Estimate	Parameter				
		β_0	β_C	β_W	β_{W2}	β_{W3}
	logit(α_G)	-2.8 (0.9)	1.6 (1.1)	-56.9 (8.5)	20.2 (8.0)	43.1 (7.5)
1	log(μ_G)	-2.7 (0.3)	-0.8 (0.4)	-3.0 (4.2)	9.0 (3.8)	13.0 (4.5)
	ω	5.9 (0.7)				
	logit(α_G)	-5.3 (1.7)	1.6 (2.3)			
2	log(μ_G)	-2.2 (0.7)	-1.9 (0.9)	-3.0 (7.7)		
	ω	28.5 (13.8)				
	logit(α_G)	-2.4 (0.2)	7.8 (0.5)	34.4 (12.0)	-5.9 (7.4)	12.1 (10.9)
3	log(μ_G)	-2.2 (0.0)		25.5 (1.7)	-11.9 (3.6)	1.8 (2.1)
	ω	2.8 (0.1)				
	logit(α_G)	-4.5 (0.3)	6.1 (0.6)	-4.5 (7.8)	-4.9 (8.7)	
4	log(μ_G)	-2.3 (0.5)	-2.1 (0.9)	-12.4 (6.1)	-2.7 (4.7)	
	ω	3.7 (0.5)				
	logit(α_G)	-2.7 (0.4)	5.4 (0.6)	-31.7 (7.4)	-48.8 (7.3)	64.8 (17.0)
5	log(μ_G)	-2.4 (0.3)	-0.6 (0.3)	-19.7 (2.8)	-12.0 (2.5)	15.4 (5.3)
	ω	2.4 (0.1)				
	logit(α_G)	-3.6 (0.3)	4.1 (0.4)	16.5 (8.6)	-61.4 (10.2)	-25.0 (10.5)
6	log(μ_G)	-3.0 (0.2)	-0.5 (0.3)	0.9 (5.7)	-39.1 (8.3)	-4.3 (6.1)
	ω	2.5 (0.2)				
	logit(α_G)	-5.4 (0.6)	6.7 (0.8)	-67.8 (21.1)	-82.8 (25.3)	
7	log(μ_G)	-3.4 (0.4)	-0.5 (0.5)	-7.1 (6.3)	-14.3 (7.0)	31.6 (9.3)
	ω	3.6 (0.4)				

Table S7. Bayesian model weights for estimating the proportion of animals in the nearshore cells that are pregnant females (p_{preg}) using logistic regression (likelihood: $n_{Preg} \sim \text{Bin}(n_{All}, p_{preg})$ where n_{All} = count of all identified individuals nearshore and n_{Preg} = count of identified pregnant females). Pregnant animals were defined as all possible pregnancies or females with known pregnancies (returned with a calf the following year). Logit(p_{preg}) was estimated as functions of visibility, Beaufort sea state, week, and week^2. Week and week^2 were orthogonally transformed to avoid correlation between variables.

	Explanatory variables				Model weights	
Model #	Visibility	Beaufort	Week	Week^2	Known pregnancies	All possible pregnancies
1					0.281	0.305
2	x				0.000	0.000
3		x			0.000	0.000
4			x		0.412	0.063
5			x	x	0.306	0.631
6	x	x			0.000	0.000
7	x		x		0.001	0.000
8	x		x	x	0.000	0.000
9		x	x		0.000	0.000
10		x	x	x	0.000	0.000
11	x	x	x		0.000	0.000
12	x	x	x	x	0.000	0.000

Table S8. Posterior distribution of parameters (β) estimating the proportion of animals in the nearshore cells that are pregnant females (p_{preg}) using logistic regression. Pregnant animals were defined as all possible pregnancies or females with known pregnancies (returned with a calf the following year). Logit(p_{preg}) was estimated as functions of visibility (V), Beaufort sea state (B), week (W), and week^2 ($W2$). Week and week^2 were orthogonally transformed to avoid correlation between variables. Visibility and Beaufort sea state were not included as variables in final model averages. Means with standard deviations in parentheses.

Parameter	Known pregnancies	All possible pregnancies
β_0	-3.38 (0.19)	-2.6 (0.13)
β_W	-10.75 (7.32)	-5.1 (4.15)
β_{W2}	0.74 (2.65)	6.5 (4.64)
